# Fluid Management Based on Bioimpedance, Blood Volume, and Patient Reports: A Quality Improvement Project in Maintenance Hemodialysis

**DOI:** 10.1016/j.xkme.2025.101217

**Published:** 2025-12-15

**Authors:** Sebastian Mussnig, Luis Naar, Simon Krenn, Florian Brosch, Daniel Schneditz, Joachim Beige, Manfred Hecking

**Affiliations:** 1 Department of Epidemiology, Center for Public Health, Medical University of Vienna, Vienna, Austria; 2 Department of Medicine III, Division for Nephrology and Dialysis, Medical University of Vienna, Vienna, Austria; 3 AIT Austrian Institute of Technology GmbH, Center for Health & Bioresources, Medical Signal Analysis; 4 IUVABIT e.U., Analytics & Data Science, Vienna, Austria; 5 Division of Physiology & Pathophysiology, Otto Loewi Research Center for Vascular Biology, Immunology and Inflammation, Medical University of Graz, Graz, Austria; 6 Kuratorium for Dialysis and Transplantation e.V. (KfH), Neu-Isenburg, Germany; 7 Martin-Luther-University Halle/Wittenberg, Medical Clinic 2, Germany

**Keywords:** Hemodialysis, fluid management, ultrafiltration, bioimpedance, blood volume, patient-reported outcome measures

## Abstract

**Rationale & Objective:**

Fluid management in hemodialysis aims to remove excess fluid while avoiding symptoms of fluid depletion. This study evaluated the impact of determining euvolemic body mass by bioimpedance spectroscopy, and absolute blood volume (ABV), on clinical practice and patient-reported outcome measures (PROMs) at a bioimpedance/ABV-naïve dialysis center.

**Study Design:**

Fourteen week quality improvement project with 3 Assessment phases separated by 2 Adjustment phases.

**Setting & Participants:**

Total of 127 patients at a single dialysis center.

**Quality Improvement Activities:**

Bioimpedance-spectroscopy-derived fluid overload (FO), ABV, and PROMs were longitudinally recorded. Physicians received data from each Assessment phase to inform treatment decisions.

**Outcomes:**

Fluid overload, ABV, PROMs, and agreement between perceived fluid status and FO.

**Analytic Approach:**

Generalized linear mixed-effects models analyzed changes over time and associations between FO, ABV, and PROMs. Agreement between perceived fluid status and FO was evaluated with linearly weighted Cohen’s κ.

**Results:**

With each Assessment phase, pre-dialysis FO, systolic and diastolic blood pressure decreased overall (−0.12 L, *P* = 0.006; −1.43 mm Hg, *P* = 0.003; −0.83 mm Hg, *P* < 0.001), with a stronger reduction in baseline fluid overloaded (FO relative to extracellular fluid >15%) patients (−0.25 L, *P* < 0.001; −1.84 mm Hg, *P* = 0.011; −1.18 mm Hg, *P* = 0.01). The difference between post-dialysis and euvolemic body mass decreased in fluid overloaded patients (−0.19 kg, *P* = 0.006). Odds of longer recovery time increased (1.43, *P* = 0.017), but no significant changes in intradialytic complications or hypotension occurred, and FO and ABV were not associated with any PROM (including recovery time) overall and separately in fluid overloaded patients. Agreement between perceived and bioimpedance-spectroscopy-derived FO was poor (κ: 0.007-0.037 for patients, 0.022-0.018 for nurses).

**Conclusions:**

During the introduction of bioimpedance- and ABV-guided fluid management at a hemodialysis center, fluid status improved without significant changes in intradialytic morbid events. The lack of agreement between perceived fluid status and bioimpedance-spectroscopy-derived FO reflects the daily clinical struggle when negotiating fluid management based on objective measures.

## Plain-language Summary

Hemodialysis allows to remove excess fluid in patients with impaired kidney function who produce little-to-no urine. Closely monitoring fluid status is necessary to minimize both fluid depletion and fluid overload. This study investigated the combination of objective and subjective modalities to inform treatment decisions through a 14-week quality improvement project at a dialysis center. By introducing 2 objective assessments of fluid status (bioimpedance spectroscopy, and absolute blood volume) coupled with repeated digital questionnaires regarding the subjective health of patients, fluid status was significantly improved. Interestingly, the subjective perception of fluid overload of patients and nurses did not agree with objective findings. Combining objective and subjective measures in an inclusive setting may be necessary when negotiating fluid management with patients.

In the absence of residual kidney function, ultrafiltration of extracellular fluid from intravascular volume is the primary mechanism for removing excess fluid.^1^ Setting ultrafiltration goals for patients on maintenance hemodialysis requires re-evaluating fluid status before each treatment in a complex, shared decision-making process that involves measured variables such as body mass and blood pressure but also feedback from patients regarding their subjective well-being and reported symptoms.^2^

Objective measures of fluid status encompass the assessment of whole-body tissue hydration via bioimpedance spectroscopy,^3^ pulmonary congestion via lung ultrasound,^4^ and intravascular volume via relative^5^ and absolute^6^ blood volume monitoring and blood pressure. Each of the corresponding measurements has been shown to be associated with mortality (higher fluid overload,^7^ higher number of lung B-lines,^8^ both intradialytic hypertension^9^ and hypotension^10^) or at least morbidity (more frequent intradialytic hypotensive events at lower specific blood volume^11^). Lung ultrasound^12^ or relative blood volume^13^ did not improve survival when used to guide fluid management, with conflicting evidence for bioimpedance-derived fluid status.^14^, ^15^, ^16^ So far, some large dialysis networks such as the German Kuratorium for Dialysis and Transplantation e.V. (KfH), currently serving roughly 18,000 dialysis patients, and entire countries such as the United States have been relatively naïve to bioimpedance-guided fluid status assessment, likely because of missing reimbursements and limited availability of bioimpedance devices.

The importance of recognizing and managing symptoms and patient-reported outcome measures (PROMs) among patients on maintenance hemodialysis was recently highlighted by a Kidney Disease: Improving Global Outcomes controversies conference.^17^ Dialysis nurses were identified for their pivotal role in managing symptoms due to their close proximity to patients.^18^ In 2006, Machek et al^19^ already used a combination of fluid overload and adverse events reported by patients and requiring nurse intervention, to guide an entire hemodialysis center toward euvolemia within a quality improvement framework. Stenberg et al^20^ coupled bioimpedance measurements with a symptom score for nurses that included signs of fluid depletion and fluid overload within a decision algorithm.

The aim of this study was to build on these previous efforts^19^^,^^20^ combining instrument-based measurements of fluid status with symptom scores and patient-reported outcomes. Specifically, we sought (I) to investigate longitudinal changes of objective measures of whole-body and intravascular fluid volume and PROMs associated with fluid status throughout a period of targeted fluid management, (II) to analyze effects of fluid status on PROMs, and (III) to describe changes in the patients’ and nurses’ perception of fluid status throughout this process.

## Materials and Methods

### Population and Ethics

In May 2024, the KfH implemented a quality improvement project at the KfH dialysis center Weiden (Weiden in der Oberpfalz, Germany) in cooperation with the Medical University of Vienna (Vienna, Austria). The aim of this project was to longitudinally assess fluid status, chronic-kidney-disease-associated pruritus, and depression in patients undergoing maintenance hemodialysis using objective measures, symptom scores, and PROMs and to adjust treatment if any measures suggested the need thereof. All patients of the KfH dialysis center Weiden treated with regular in-center maintenance hemodialysis were included in the project. The quality improvement project was authorized by the institutional review board of the KfH with a requirement to process pseudonymized data internally only. In addition, the requirement for an ethics review before the retrospective analysis and publication was waived by the ethics committee of the Bavarian State Medical Association after a formal request. The methodology used in the project was part of routine clinical practice, did not pose additional risks and was therefore conducted without formal written informed consent. Data on pruritus and depression have been analyzed separately and were in submission for publication when the present manuscript was accepted (Naar et al., Journal of Patient-Reported Outcomes).

### Design

**Figure 1:**
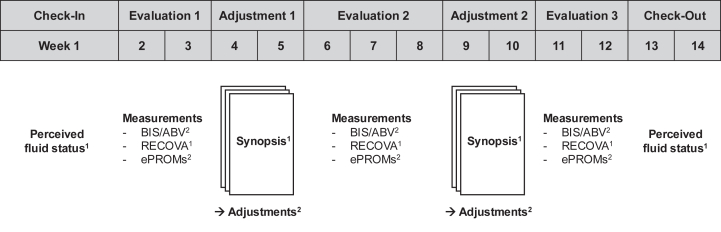
Flowchart of the quality improvement project. The quality improvement project lasted 14 weeks. Patients and nurses reported how they perceived the patient’s fluid status during *Check-In* (week 1) and *Check-Out* (weeks 13-14). Electronic patient-reported outcome measures (ePROMs) were captured at each dialysis treatment between weeks 2-12. Bioimpedance-spectroscopy-(BIS)-derived fluid overload and body composition, and absolute blood volume (ABV) estimations were acquired at each treatment during *Evaluation* phases. The RECOVA symptom score was recorded once per *Evaluation* and *Adjustment* phase. Data were algorithmically summarized prior to each *Adjustment* phase to single-page synopses. Subsequent treatment adjustments were guided by these summaries. Variables with superscript (^1^) were recorded once per phase, while variables with superscript (^2^) were recorded during each treatment of the respective phase. ABV, absolute blood volume estimated by the dialysate-bolus method; BIS, bioimpedance spectroscopy; ePROMs, electronically-captured patient-reported outcome measures; RECOVA, Recognition and Correction of Volume Alterations.

The quality improvement project flowchart is shown in Fig 1. We distinguished between *Check-In*, *Evaluation*, *Adjustment*, and *Check-Out* phases.

Check-In: during week 1, patients were introduced to the project scope and were asked how they perceived their fluid status (Table S1). At least one nurse per patient was asked to evaluate the respective patient’s fluid status as well (Table S2).

Evaluation: During weeks 2-3, 6-8, and 11-12, patients underwent repeated measurements of fluid status and blood volume. Nurses were instructed to determine a symptom score for fluid status for each patient once during each *Evaluation* and *Adjustment* phase (Table S3). Patients were asked to complete a questionnaire after each dialysis treatment across all *Evaluation* and *Adjustment* phases that included recovery time, well-being, and intradialytic morbid events (Table S4).

Adjustment: During weeks 4-5 and 9-10, data previously collected within the project were compiled in single-sheet reports on each patient (Fig S1). These summaries included longitudinal visualization of body masses, fluid status, blood volume, blood pressure, intradialytic morbid events, and the symptom score throughout the entire project, and algorithmized summaries with treatment recommendations of the previous *Evaluation* phase. A euvolemic body mass corridor was computed for each *Evaluation* phase from the average pre-dialysis euvolemic body mass and ± 7% of the average extracellular fluid volume. Reports were handed to the physicians, who were free to adhere to these recommendations, to implement their own adjustment, or to continue treatment unchanged.

Check-Out: during weeks 13-14, patients and nurses were again asked about their respective perceptions of the patients’ fluid status (Tables S5 and S6).

### Procedures

Dialysis treatment: maintenance hemodialysis or hemodiafiltration treatment was prescribed at the discretion of the physicians. The DBB-EXA (37 devices), DBB07 (10 devices), and DBB05 (1 device) dialysis machines (Nikkiso Co, Ltd, Tokyo, Japan) were used for treatment.

Body composition: fluid status was measured using Multiscan 5000 (Bodystat Ltd., Douglas, Isle of Man) bioimpedance spectroscopy devices. Measurements were recommended to be made pre-dialysis in supine wrist-to-ankle electrode configurations contralateral to the vascular access side. However, data was frequently captured intra-dialysis instead. In these cases, pre-dialysis and post-dialysis body composition was approximated according to Equations 1-14 (Item S1: Supplemental Methods). Fluid overload was subsequently adjusted for age according to Equation 15 (Item S2: Supplemental Methods).

Blood volume: pre-dialysis blood volume was estimated using a dialysate-bolus method described elsewhere.^6^ Briefly, 240 mL of ultrapure dialysate were infused 60 minutes after treatment start in post-dilution mode. Blood volume could only be estimated for treatments conducted with DBB-EXA machines equipped with blood volume monitors (37 out of 48 dialysis machines). Relative blood volume before and after bolus infusion required for absolute blood volume estimation was modeled using a previously published algorithm.^21^ All estimates were excluded if data were considered implausible.

Questionnaires: surveys on fluid status perception, subjective well-being, dialysis quality, intradialytic morbid events, and the Recognition and Correction of Volume Alterations (RECOVA) symptom score^20^ (Tables S1-S6) were conducted digitally (Typeform S.L., Barcelona, Spain) in German. Patients and nurses accessed the surveys either directly through Typeform via a facility tablet or through the Mizu smartphone application (Carealytix Digital Health GmbH, Hohendilching, Germany) that had to be downloaded and set up on the patients’ personal devices.

Ambulatory blood pressure: arterial blood pressure was measured by arm-cuff and oscillometric technique pre-dialysis, post-dialysis, and in varying frequencies intra-dialysis according to the center’s common practice. Intradialytic hypotension was defined by the Nadir 90/100 criteria.^10^

### Statistical Analysis

For summary tables, continuous variables were summarized as means of patient means (standard deviation of patient means) per stratum, while nominal and ordinal variables were described using counts and relative frequencies for each level. Changes of continuous variables over time were depicted as line plots with bootstrapped 95% confidence intervals (CI), and as column plots for binary and ordinal variables overall and stratified by the average pre-dialysis relative fluid overload during *Evaluation 1* (<7%, 7%-15%, or >15%).

Time-course analysis: changes across *Evaluation* phases were investigated by fitting linear mixed-effects models for continuous dependent variables (DVs), cumulative link mixed models for ordinal DVs and generalized linear mixed-effects models with binomial link functions for binary DVs. Model parameters were reported as fixed effect estimates with 95% CI for continuous DVs and odds ratios (OR) with 95% CI for ordinal and binary DVs. Analyses were restricted to data from *Evaluation* phases, with the independent variable of interest (IVOI) being either day (within the project) or phase (*Evaluation* phases 1-3) added to the model using fixed effects. Additional independent variables with fixed effects included baseline variables age (z-score), sex, type 2 diabetes mellitus, heart failure, albumin concentration (z-score), hemoglobin concentration (z-score), and dialysis vintage (z-score). Random intercepts were included for each patient, and random slopes for day or phase were added if model convergence permitted. Details on variable preparation are provided in Table S7. We defined 3 analyses: the complete-cases analysis consisted of patients who had at least one observation of the DV during each of the 3 *Evaluation* phases. The fluid-overloaded analysis had the same criteria as the complete-cases analysis, but only patients with average relative fluid overload >15% during *Evaluation* 1 were investigated. The available-cases analysis included all patients who had at least one observation of the DV, regardless of the completeness across *Evaluation* phases.

Short-term effects: the delayed effects of post-dialysis relative fluid overload and blood volume on variables from the subsequent treatment were analyzed using models chosen based on the variable type, as described above. The delay for the IVOI was set at 2-3 days, corresponding to the interdialytic interval. These models included fixed effects for the IVOI, an interaction term between the IVOI and the time delay, confounders as described above, random intercepts for each patient, and random slopes for the IVOI if model convergence permitted. Analyses were computed across all patients and in patients with average relative fluid overload >15% during *Evaluation* 1, regardless of completeness across phases.

Perceived and measured fluid status: pre-dialysis relative fluid overload averaged over weeks 2-3 was compared with perceived fluid status from *Check-In* (week 1), and pre-dialysis relative fluid overload averaged over weeks 11-12 was compared with perceived fluid status from *Check-Out* (week 13-14). Fluid overload was categorized as pre-dialysis relative fluid overload <7%, 7%-15%, or >15%, and perceived fluid status was categorized as fluid depleted, euvolemic, and fluid overloaded. Linearly weighted Cohen’s κ was calculated for both timepoints, each between patients and nurses, between patients and bioimpedance, and between nurses and bioimpedance, to assess the reliability of classification. Only complete cases (i.e., completeness of data from *Check-In* and *Check-Out* for patient perception, nurse perception and bioimpedance) were used for these analyses.

P-values across all analyses were corrected to control the false discovery rate via the Benjamini-Hochberg procedure. Adjusted *P*-values were evaluated against the significance level of α = 0.05. Model fits were investigated by conditional coefficients of determination (R^2^) and visual inspection of model residuals. All statistical analyses were carried out using R programming language version 4.4.1 and RStudio for macOS version 2024.09.0+375 (Posit Software).

## Results

### Population Characteristics and Data Completeness

At the beginning of May 2024, 133 patients were undergoing regular in-center maintenance hemodialysis treatment at the KfH dialysis center Weiden, of whom 127 patients contributed data during the quality improvement period between May 27 and September 14, 2024. The mean age and dialysis vintage were 69 (± 14) years and 59 (± 61) months, respectively, and 69% of patients were men (Table 1).

**Table 1:** Baseline Patient Characteristics Overall and Stratified by Relative Fluid Overload

**Variable**	**N**	Overall	<7% n = 16	7%-15% n = 46	>15% n = 48
**Age, y**	127	69 (14)	71 (13)	68 (14)	69 (14)
**Sex**	127				
Female		39 (31%)	4 (25%)	13 (28%)	16 (33%)
Male		88 (69%)	12 (75%)	33 (72%)	32 (67%)
**Dialysis modality**	119				
Hemodiafiltration		6 (5.0%)	2 (13%)	1 (2.2%)	3 (6.5%)
Hemodialysis		113 (95%)	14 (88%)	44 (98%)	43 (93%)
**Dialysis vintage, mo**	126	56 (61)	33 (18)	49 (43)	63 (76)
**Residual diuresis, mL**	72	636 (552)	681 (607)	629 (484)	609 (568)
**Cause of kidney failure**	113				
Diabetes		25 (22%)	1 (7.7%)	4 (10%)	14 (32%)
Glomerular disease		45 (40%)	5 (38%)	16 (41%)	15 (34%)
Vascular disease		16 (14%)27 (24%)	4 (31%)	7 (18%)12 (31%)	10 (23%)
Other		16 (14%)	3 (23%)	7 (18%)	5 (11%)
**History of kidney transplant**	127	6 (4.7%)	1 (6.3%)	3 (6.5%)	2 (4.2%)
**Type 2 diabetes mellitus**	127	47 (37%)	1 (6.3%)	17 (37%)	23 (48%)
**Heart failure (HFrEF or HFpEF)**	127	36 (28%)	1 (6.3%)	13 (28%)	18 (38%)
**Arterial hypertension**	127	102 (80%)	12 (75%)	38 (83%)	37 (77%)
**Coronary artery disease**	127	51 (40%)	5 (31%)	14 (30%)	24 (50%)
**History of stroke**	127	16 (13%)	2 (13%)	8 (17%)	3 (6.3%)
**Albumin, g/dL**	118	3.61 (0.41)	3.67 (0.38)	3.66 (0.40)	3.53 (0.42)
**Hemoglobin, g/L**	118	114 (15)	117 (12)	115 (13)	112 (18)
**Antihypertensive medications**	127				
0		71 (56%)	11 (69%)	26 (57%)	26 (54%)
1		35 (28%)	4 (25%)	15 (33%)	10 (21%)
2		6 (4.7%)	0 (0%)	2 (4.3%)	2 (4.2%)
3		7 (5.5%)	0 (0%)	1 (2.2%)	6 (13%)
4 or more		8 (6.3%)	1 (6.3%)	2 (4.3%)	4 (8.3%)

*Note:* The data are reported as frequency (percentage) or mean (standard deviation) overall and stratified by average relative fluid overload during *Evaluation* 1 (<7%, 7%-15%, and >15%). Note that fluid overload was not available for all patients during *Evaluation* 1, thus the stratified cohort may be smaller than the overall cohort. HFrEF, heart failure with reduced ejection fraction; HFpEF, heart failure with preserved ejection fraction.

### Adherence to the Quality Improvement Schedule

The proportion of patients who recorded PROMs and who had valid absolute blood volume estimations at least once per *Evaluation* phase decreased throughout the project (PROMs: 86% to 80% to 59%; blood volume: 80% to 76% to 61%), whereas the proportion of patients who received at least one bioimpedance assessment per *Evaluation* phase remained stable (90% to 92% to 87%) (Table S8). Seventy-five patients owned a smartphone compatible with the Mizu application, 33 of whom were able to set up the user profile correctly. Seventy-five percent of all PROM captures required assistance from the nurses, and 21 patients (17.5% of patients who provided PROMs) required no assistance at all.

### Time Course of Analyzed Variables

**Figure 2:**
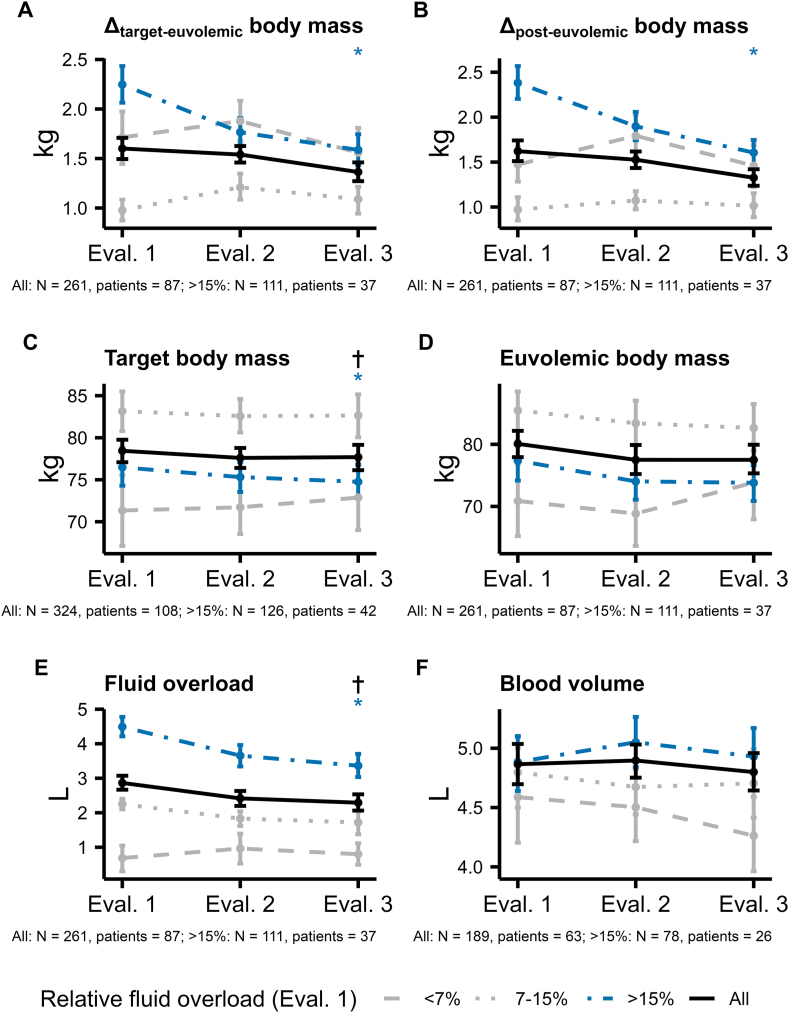
Continuous variables throughout *Evaluation* phases overall and stratified by relative fluid overload. The data are shown as mean (dots) and bootstrapped 95% confidence interval of the mean (error bars) overall (solid black line) and stratified by average relative fluid overload during *Evaluation* 1 (<7%: gray dashed line; 7%-15% gray dotted line; >15%: blue dash-dotted line). † and ∗ denote whether the variable significantly changed throughout the project in the complete-cases and fluid-overloaded analysis, respectively. Values below the subpanels indicate the number of observations and patients used in the generalized linear mixed-effects models and for visualization. Note that fluid overload was not available for all patients during *Evaluation* 1, thus the stratified cohort may be smaller than the overall cohort.

**Figure 3:**
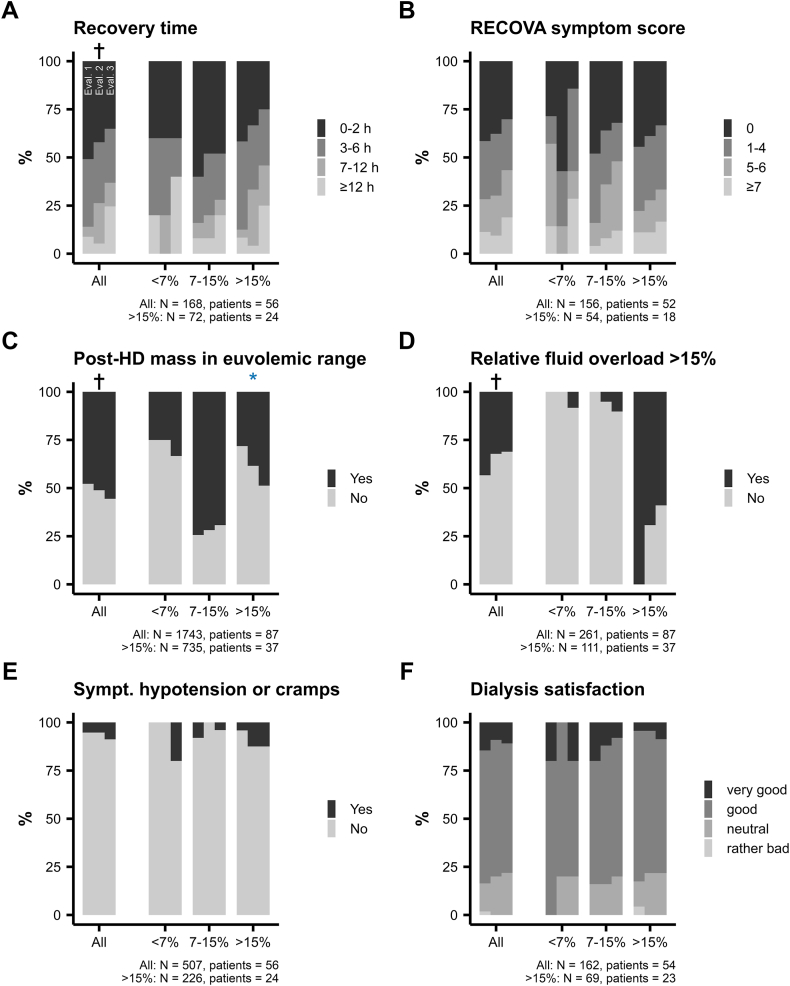
Ordinal and nominal variables throughout *Evaluation* phases overall and stratified by relative fluid overload. The data are displayed as percentages per level across *Evaluation* phases (see Panel A, *Evaluation* 1: left sub-column; *Evaluation* 2: middle sub-column; and *Evaluation* 3: right sub-column overall (“All”) and stratified by average relative fluid overload during *Evaluation* 1 (“<7%”, “7%-15”, >15%). † and ∗ denote whether the variable significantly changed throughout the project in the complete-cases and fluid-overloaded analysis, respectively. Values below the subpanels indicate the number of observations and patients used in the generalized linear mixed-effects models and for visualization. Note that fluid overload was not available for all patients during *Evaluation* 1, thus the stratified cohort may be smaller than the overall cohort. HD, hemodialysis; sympt., symptomatic.

Data summarized by *Evaluation* phase are listed in Table 2 and Table S9. Model estimates for the complete-cases, fluid-overloaded and available-cases analyses are listed in Table 3, Table 4 and Table S10, and selected variables are depicted throughout *Evaluation* phases overall and stratified by relative fluid overload in Figs 2 and 3. Euvolemic body mass did not change throughout the project in any analysis, but metrics of fluid overload, pre-dialysis systolic and diastolic blood pressure decreased over time. Overall, the difference from euvolemic body mass to post-dialysis or target body mass did not change, but a significant reduction was observed in fluid overloaded patients. The number of prescribed antihypertensive medications and recovery time increased throughout the project in the complete-cases and available-cases analyses, but not in fluid overloaded patients. Odds of symptomatic hypotension or cramps, intradialytic hypotension, or symptoms of fluid overload or depletion summarized by the RECOVA symptom score did not change over time in any analysis. Run charts of selected parameters are shown in Fig S2.

**Table 2:** Data Throughout *Evaluation* Phases

**Variable**	**Obs**	***Evaluation* 1 (week 2-3),** n = 706	***Evaluation* 2** (week 6-8), n = 1,006	*Evaluation* 3 (week 11-12), n = 717
**Body composition**				
Pre-dialysis body mass, kg	360	79.95 (17.93)	79.45 (17.89)	79.15 (17.94)
Post-dialysis body mass, kg	358	77.83 (17.55)	77.50 (17.51)	77.23 (17.49)
Target body mass, kg	358	77.12 (17.37)	76.87 (17.33)	76.75 (17.33)
Euvolemic body mass, kg	320	77.04 (18.07)	76.86 (18.09)	76.53 (18.35)
Lean tissue mass, kg	320	39.71 (10.60)	41.23 (10.58)	41.41 (11.70)
Adipose tissue mass, kg	320	37.33 (16.90)	35.63 (17.38)	35.12 (17.67)
Target body mass in euvolemic range	2,103	327 (53%)	450 (49%)	288 (50%)
Post-dialysis body mass in euvolemic range	2,103	309 (50%)	427 (47%)	311 (54%)
Δ target-euvolemic body mass, kg	320	1.59 (1.28)	1.65 (1.34)	1.52 (1.29)
Δ post-dialysis-euvolemic body mass, kg	320	1.64 (1.22)	1.68 (1.27)	1.48 (1.22)
Body mass index, kg/m^2^	360	27.10 (5.20)	26.90 (5.20)	26.77 (5.22)
**Fluid volumes**				
Cumulative ultrafiltration volume, L	358	2.55 (1.18)	2.61 (1.17)	2.53 (1.14)
Pre-dialysis fluid overload, L	320	2.91 (1.80)	2.72 (1.79)	2.58 (1.85)
Pre-dialysis relative fluid overload, %	320	14 (8)	13 (8)	13 (8)
Post-dialysis fluid overload, L	320	0.35 (1.87)	0.15 (1.92)	0.03 (1.80)
Post-dialysis relative fluid overload, %	320	1 (11)	0 (12)	-1 (10)
Pre-dialysis blood volume, L	261	5.26 (1.08)	5.27 (1.09)	5.14 (1.03)
Post-dialysis blood volume, L	261	4.86 (1.04)	4.88 (1.08)	4.75 (0.99)
Pre-dialysis specific blood volume, mL/kg	261	69 (16)	69 (14)	68 (13)
Post-dialysis specific blood volume, mL/kg	261	64 (15)	64 (14)	63 (13)
**Blood pressure**				
Pre-dialysis systolic blood pressure, mm Hg	358	135 (19)	131 (19)	129 (20)
Pre-dialysis diastolic blood pressure, mm Hg	358	73 (12)	71 (12)	69 (11)
Intradialytic hypotension (Nadir 90/100)	1,988	37 (5.8%)	79 (9.6%)	59 (11%)
Symptomatic intradialytic hypotension	758	17 (5.2%)	16 (5.4%)	12 (8.8%)
Symptomatic intradialytic hypertension	758	10 (3.0%)	7 (2.4%)	5 (3.7%)

*Note:* The data are reported as means of patient means (standard deviation of patient means) or frequency (percentage) of available data and are stratified by *Evaluation* phase. Specific blood volume was normalized to post-dialysis body mass. Not all data were available from all dialysis treatments, hence, the number of observations per variable are lower than the total number of all studied treatments (N).

**Table 3:** Parameter Estimates of Mixed-Effects Models for Day or Phase Within the Project From the Complete-Cases Analysis

DV	IVOI	β_ivoi_ [95% CI]	*P*_ivoi_	σ_0_	σ_1_	N	Patients	R^2^
**Body composition**								
Pre-dialysis body mass	Phase	−0.21 [−0.3 to −0.12]	< 0.001	16.29	0.41	324	108	1.00
Post-dialysis body mass	Phase	−0.21 [−0.3 to −0.11]	< 0.001	15.97	0.43	324	108	1.00
Target body mass	Phase	−0.17 [−0.26 to −0.08]	0.002	15.89	0.43	324	108	1.00
Euvolemic body mass	Phase	−0.05 [−0.15 to 0.06]	0.59	17.38	0.40	261	87	1.00
Lean tissue mass	Phase	0.58 [0.32-0.85]	< 0.001	7.31	0.55	261	87	0.92
Adipose tissue mass	Phase	−0.63 [−0.89 to −0.36]	< 0.001	17.07	0.58	261	87	0.97
Target body mass in euvolemic range∗	Phase	0.83 [0.55-1.27]	0.60	7,438.13	3.61	261	87	0.85
Post-dialysis body mass in euvolemic range∗	Day	1.01 [1-1.01]	0.002	10.77	NA	1,743	87	0.64
Δ target—euvolemic body mass	Phase	−0.05 [−0.12 to 0.02]	0.30	1.35	0.21	261	87	0.66
Δ post-dialysis—euvolemic body mass	Phase	−0.06 [−0.13 to 0]	0.17	1.30	0.22	261	87	0.72
**Fluid volumes**								
Pre-dialysis fluid overload	Phase	−0.12 [−0.2 to −0.05]	0.006	1.62	0.28	261	87	0.88
Pre-dialysis relative fluid overload	Phase	−0.6 [−0.91 to −0.29]	0.002	6.82	1.10	261	87	0.87
Relative fluid overload >15%∗	Phase	0.68 [0.51-0.92]	0.04	92.41	NA	261	87	0.88
Post-dialysis fluid overload	Phase	−0.13 [−0.2 to −0.05]	0.008	1.91	0.27	261	87	0.85
Post-dialysis relative fluid overload	Phase	−0.7 [−1.14 to −0.26]	0.01	11.72	1.49	261	87	0.86
Pre-dialysis blood volume	Phase	−0.02 [−0.07 to 0.03]	0.59	0.81	0.02	189	63	0.78
**Blood pressure**								
Pre-dialysis systolic blood pressure	Phase	−1.43 [−2.21 to −0.65]	0.003	19.68	2.77	324	108	0.81
Pre-dialysis diastolic blood pressure	Phase	−0.83 [−1.22 to −0.45]	< 0.001	10.76	1.12	324	108	0.83
Intradialytic hypotension∗	Day	1 [0.99-1.02]	0.81	4.09	1.03	1,784	101	0.39
Number of antihypertensive medications∗	Phase	1.3 [1.08-1.56]	0.02	1,612.07	NA	327	109	0.95
**Patient-reported outcomes**								
RECOVA symptom score∗	Phase	1.23 [1.02-1.48]	0.09	2.18	1.04	156	52	0.31
Recovery time∗	Phase	1.43 [1.12-1.83]	0.02	12.67	1.05	168	56	0.68
Dialysis satisfaction∗	Phase	0.97 [0.76-1.24]	0.90	11.05	NA	162	54	0.66
Well-being∗	Phase	0.87 [0.66-1.15]	0.56	18.30	1.94	168	56	0.65
Symptomatic hypotension or cramps∗	Day	1.04 [0.99-1.09]	0.24	27.10	1.03	507	56	0.66

*Note:* Data from patients who had at least one observation during each of the 3 *Evaluation* phases for the dependent variable (DV) were included in this analysis. DV were modeled with generalized linear mixed-effects models, with either project day or *Evaluation* phase as the independent variable of interest (IVOI). Models included baseline age (z-score), sex, type 2 diabetes mellitus, heart failure, albumin concentration (z-score), hemoglobin concentration (z-score) and dialysis vintage (z-score) as covariates, “$\beta_{ivoi}$ [95% CI]” denotes the fixed effect estimate of the IVOI. Model parameters of DVs marked with an asterisk (∗) were reported as odds ratios, and as regression coefficients otherwise. $\sigma_{0}$ and $\sigma_{1}$ indicate the standard deviation of intercepts and slopes between patients. If $\sigma_{1}$ is NA, the model was fit without random slopes. CI, confidence interval; DV, dependent variable; IVOI, independent variable of interest; SD, standard deviation.

**Table 4:** Parameter Estimates of Mixed-Effects Models for Day or Phase Within the Project in Fluid Overloaded Patients

DV	IVOI	β_ivoi_ [95% CI]	*P*_ivoi_	σ_0_	σ_1_	N	Patients	R^2^
**Body composition**								
Pre-dialysis body mass	Phase	−0.38 [−0.55 to −0.21]	< 0.001	15.66	0.43	126	42	1.00
Post-dialysis body mass	Phase	−0.4 [−0.57 to −0.23]	< 0.001	15.49	0.46	126	42	1.00
Target body mass	Phase	−0.37 [−0.52 to −0.21]	< 0.001	15.62	0.47	126	42	1.00
Euvolemic body mass	Phase	−0.08 [−0.25 to 0.09]	0.57	15.84	0.44	111	37	1.00
Lean tissue mass	Phase	0.44 [0.09-0.79]	0.05	8.08	0.73	111	37	0.96
Adipose tissue mass	Phase	−0.52 [−0.9 to −0.15]	0.03	14.22	0.77	111	37	0.98
Target body mass in euvolemic range∗	Phase	1.59 [0.91-2.79]	0.25	304.66	2.25	111	37	0.74
Post-dialysis body mass in euvolemic range∗	Day	1.03 [1.02-1.04]	< 0.001	6.90	NA	735	37	0.62
Δ target—euvolemic body mass	Phase	−0.17 [−0.28 to −0.06]	0.02	1.54	0.24	111	37	0.72
Δ post-dialysis—euvolemic body mass	Phase	−0.19 [−0.29 to −0.08]	0.006	1.50	0.26	111	37	0.86
**Fluid volumes**								
Pre-dialysis fluid overload	Phase	−0.25 [−0.35 to −0.15]	< 0.001	1.22	0.25	111	37	0.89
Pre-dialysis relative fluid overload	Phase	−1.14 [−1.55 to −0.74]	< 0.001	3.47	0.92	111	37	0.85
Relative fluid overload >15%∗	Phase	0.17 [0.01-2.54]	0.39	70.97	NA	111	37	0.91
Post-dialysis fluid overload	Phase	−0.29 [−0.4 to −0.18]	< 0.001	1.38	0.27	111	37	0.86
Post-dialysis relative fluid overload	Phase	−1.66 [−2.19 to −1.14]	< 0.001	6.79	NA	111	37	0.79
Pre-dialysis blood volume	Phase	−0.01 [−0.08 to 0.06]	0.89	0.95	NA	78	26	0.80
**Blood pressure**								
Pre-dialysis systolic blood pressure	Phase	−1.84 [−3 to −0.69]	0.01	13.61	NA	126	42	0.69
Pre-dialysis diastolic blood pressure	Phase	−1.18 [−1.89 to −0.47]	0.01	11.07	1.63	126	42	0.83
Intradialytic hypotension∗	Day	0.99 [0.97-1.01]	0.76	3.87	1.04	691	39	0.35
Number of antihypertensive medications∗	Phase	2.17 [1.13-4.18]	0.06	563,775.57	3.25	126	42	0.98
**Patient-reported outcomes**								
RECOVA symptom score∗	Phase	1.23 [0.88-1.73]	0.43	2.62	1.06	54	18	0.40
Recovery time∗	Phase	1.29 [0.94-1.77]	0.26	3.33	1.15	72	24	0.53
Dialysis satisfaction∗	Phase	0.82 [0.44-1.53]	0.72	4,527.15	1.66	69	23	0.93
Well-being∗	Phase	0.91 [0.62-1.36]	0.80	5.57	1.70	72	24	0.42
Symptomatic hypotension or cramps∗	Day	1.02 [1-1.04]	0.08	5.22	NA	226	24	0.53

*Note:* Data from patients with average relative fluid overload >15% during *Evaluation* 1 who had at least one observation during each of the 3 *Evaluation* phases for the dependent variable (DV) were included in this analysis. DV were modeled with generalized linear mixed-effects models, with either project day or *Evaluation* phase as the independent variable of interest (IVOI). Models included baseline age (z-score), sex, type 2 diabetes mellitus, heart failure, albumin concentration (z-score), hemoglobin concentration (z-score) and dialysis vintage (z-score) as covariates, “$\beta_{ivoi}$ [95% CI]” denotes the fixed effect estimate of the IVOI. Model parameters of DVs marked with an asterisk (∗) were reported as odds ratios, and as regression coefficients otherwise. $\sigma_{0}$ and $\sigma_{1}$ indicate the standard deviation of intercepts and slopes between patients. If $\sigma_{1}$ is NA, the model was fit without random slopes. CI, confidence interval; DV, dependent variable; IVOI, independent variable of interest; SD, standard deviation.

### Short-Term Effects of Fluid Overload and Blood Volume

Neither post-dialysis fluid overload nor post-dialysis blood volume showed a significant association with recovery time, well-being, dialysis satisfaction, the RECOVA symptom score or the number of antihypertensive medications both in the overall and in the fluid overloaded population (Table S11 and Table S12). Post-dialysis fluid overload showed no association with pre-dialysis blood volume of the subsequent treatment.

### Perception of Fluid Status and Project Acceptance

**Figure 4:**
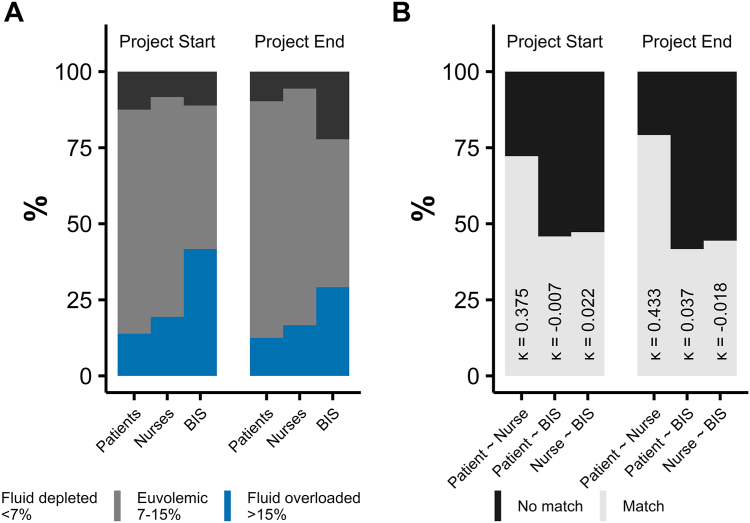
Perceived and bioimpedance-derived fluid status and agreement at project start and end. Panel A shows the fluid status as perceived by patients and nurses and as indicated by bioimpedance at project start (perceived: during week 1; bioimpedance: averaged over weeks 2-3) and project end (perceived: during weeks 13-14; bioimpedance: averaged over weeks 11-12). Panel B depicts the agreements of classification between patients, nurses and bioimpedance for both timepoints. κ values indicate the linearly weighted Cohen’s κ for agreements.

Categorizations and agreements between patients, nurses and bioimpedance measurements regarding fluid status are shown in Fig 4. At *Check-In* and *Check-Out*, patients and nurses agreed moderately (72.0% and 79.2%), whereas neither patients (45.8% and 41.7%) nor nurses (47.2% and 44.4%) agreed with the bioimpedance-derived classification. Twenty-two percent of patients felt that procedures for fluid status assessment within the project required too much effort. The proportion of patients and nurses who considered fluid status assessment to be adequate increased from *Check-In* to *Check-Out* (patients: 58% to 75%; nurses: 69% to 76%; Table S13).

## Discussion

Here, we present novel insights into the response of longitudinal whole-body fluid overload, absolute blood volume, and PROMs to changes in fluid management among patients undergoing maintenance hemodialysis. Our quality improvement project led to an overall reduction in body mass and allowed bringing both fluid overloaded and fluid depleted patients closer to their respective euvolemic target ranges, while avoiding increases in intradialytic hypotensive events or other intradialytic symptomatic complications overall and in the fluid overloaded population. Relative fluid overload decreased, and absolute blood volume remained unchanged despite a concomitant decrease in fluid overload, potentially indicating a long-term adaptation to improved volume control. The observed increase in recovery time predominantly occurred in patients who were fluid overloaded at project start (albeit not significantly). However, changes in fluid overload were not associated with PROMs, suggesting that fluid overloaded patients may be more vulnerable to deterioration in perceived recovery even if fluid overload may not have been the culprit.

### Response of Recovery Time to Changes in Fluid Management

Rayner et al^22^ investigated associations of recovery time with clinical parameters in a large cohort of more than 6,000 patients on maintenance hemodialysis, of whom 32% reported a recovery time of <2 hours, 41% 2-6 hours, 17% 7-12 hours, and 10% >12 hours. Compared with their distribution, recovery time results reported by our patients were skewed toward shorter recovery times in the first *Evaluation* phase, and toward longer recovery times in the third *Evaluation* phase. Older age, higher dialysis vintage, body mass index, and intradialytic weight loss (i.e., ultrafiltration volume) were identified as predictors for longer recovery time.^22^ The literature showing benefits of frequent dialysis treatment on recovery time is extensive.^23^, ^24^, ^25^, ^26^ Jaber et al^23^ explored the effect of 6-times weekly hemodialysis on post-dialysis recovery time, which decreased drastically in their per-protocol analysis (476 minutes at baseline, 62 minutes after 4 months). Our patients all underwent regular thrice-weekly hemodialysis treatments. Although recovery time significantly increased throughout the quality improvement project in our cohort, neither post-dialysis fluid overload nor blood volume were associated with recovery time. The RECOVA symptom score, which itemizes symptoms both related to fluid overload and fluid depletion, did not change throughout the project and was also unaffected by fluid overload and blood volume. Patients with relative fluid overload >15% at the beginning, of whom nearly half were below 15% at the end of the quality improvement project, still experienced the most apparent increase in recovery time. Our results suggest that fluid expanded patients may be at risk of perceived health deterioration irrespective of fluid status, thereby preventing adjustment in the absence of objective measures of fluid status.

### Feasibility of Patient-Initiated Electronic PROMs

We expected that only 50% of patients would be able to track PROMs autonomously with their personal smartphones (ePROMs), but the final percentage was even lower, and we experienced that patients were frequently dependent on assistance from the staff to record symptoms. Pittman et al^27^ described daily capture of PROMs delivered through a website directed at patients. They preselected hemodialysis, peritoneal dialysis, and chronic kidney disease patients who stated to have regular access to the internet. Of 84 patients identified and included, only 43 patients provided at least one set of data. These results are similar to ours in terms of the conversion rate from identified potential users to active users. Flythe et al^28^ analyzed the feasibility of tablet-based patient-reported outcomes in a quality improvement setting similar to ours. Their ePROM tool was based on facility-issued tablets as opposed to a hybrid solution of patient smartphones and facility tablets in our study. The attrition rate varied between 70% and 97% for daily ePROM capture in their project, which was substantially higher than what we achieved with our ePROM tracking, but was similarly successful compared to our recordings of the RECOVA symptom score once per phase. Hence, we believe that symptom tracking should continue to be conducted by the medical staff and should not be passed on to patients exclusively, ensuring broad coverage of the entire hemodialysis population. A shift toward symptom tracking via the patients’ private devices may alienate, disadvantage, and exclude patients who are unable to do so due to age, language barriers or socioeconomic status.

### Discrepancies in Perceived Euvolemia

The nurses’ perception of fluid status evolved from the beginning to the end of the project to match the patients’ perception rather than the category of fluid status indicated by bioimpedance. Quality improvement data were openly communicated with the center’s team, and this mismatch between subjective and objective fluid volume assessment appears to show a rejection of bioimpedance results. However, estimates of fluid overload were not corrected for timepoint of measurement within the treatment and were not age-corrected during the active quality improvement project (i.e., physicians were handed unadjusted values), thereby limiting their usefulness and potentially explaining the large discrepancies between the patients’ and nurses’ classification of fluid status compared to bioimpedance results. The encountered skepticism is supported by a systematic bias in bioimpedance-spectroscopy-derived fluid overload especially in patients with very high or very low body mass indices.^29^ The quest toward euvolemia constitutes a balancing act between competing risks, because both intradialytic hypotension^10^ and chronic fluid overload^30^ are associated with mortality. Our data may provide a window into functional fluid overload, i.e.*,* a state of objective fluid overload that may not be noticed by patients, but that may be required to maintain hemodynamic stability, hence the mismatch.

### Prescription of Antihypertensive Medications

The increase in the number of prescribed antihypertensive medications was an unfortunate observation, which was most obvious in the fluid overloaded population. However, neither post-dialysis fluid overload nor post-dialysis blood volume were associated with the number of antihypertensive medications, suggesting that blood pressure treatment may have been prescribed independently of fluid status. Before commencing the project, we expected to observe an overall decrease in the usage of pharmacological blood pressure management in favor of increased ultrafiltration, especially in fluid overloaded patients in whom increased fluid removal could have improved both blood pressure and fluid status.

### Strengths and Limitations

This study is limited by the lack of a control arm of the underlying quality improvement project. The small sample size of the fluid overloaded population does not permit generalization to other populations, and findings must be reproduced in larger trials. A strength of the study is the broad inclusion of patients from an entire hemodialysis center, allowing valuable insights into both objective measures of fluid status and the adoption of ePROMs. The team of physicians changed halfway through the quality improvement period, possibly affecting the continuity of treatment goals throughout the entirety of the project, including the prescription of antihypertensive medications. This project was implemented as a quality improvement initiative by the KfH medical board with the intention of benefitting as many patients as possible, but we acknowledge that future studies should be conducted within a (randomized) prospective interventional framework.

### Conclusion

We observed significant improvements in fluid status without an increase in frequency of intradialytic morbid events at a bioimpedance-naïve dialysis center within three months of quality improvement measures involving longitudinal assessment of fluid overload, blood volume, and ePROMs. Adoption of electronic symptom tracking using the patients’ personal devices fell short of expectations. The lack of agreement between perceived fluid status and bioimpedance-derived fluid overload reflects the daily clinical struggle when negotiating fluid management based on objective measures.
