# Supplementary material for: Fluid Management Based on Bioimpedance, Blood Volume, and Patient Reports: A Quality Improvement Project in Maintenance Hemodialysis
Source: Kidney Med. 2025 Dec 15;8(2):101217. doi: 10.1016/j.xkme.2025.101217 (PMC12856452; doi:10.1016/j.xkme.2025.101217)
Supplement: Supplementary File (PDF) — Figures S1-S2; Methods S1-S2; Tables S1-S13. [file mmc1.pdf]

Fluid management based on bioimpedance, blood volume and patient reports: A quality improvement project in maintenance hemodialysis

Sebastian Mussnig, Luis Naar, Simon Krenn, Florian Brosch, Daniel Schneditz, Joachim Beige, Manfred Hecking

Correspondence to:
Manfred Hecking (manfred.hecking@meduniwien.ac.at)
Medical University of Vienna
Department of Medicine III, Division for Nephrology and Dialysis
Waehringer Guertel 18-20
1090 Vienna, Austria

Running head: Longitudinal objective and patient-reported fluid status in hemodialysis

Table of contents:

p.2 Supplemental Methods 1: Estimation of pre-dialysis body composition
p.4 Supplemental Methods 2: Age correction of fluid overload
p. 6 Table S1: Questionnaire for patients at *Check-In*
p. 8 Table S2: Questionnaire for nurses at *Check-In*
p. 10 Table S3: Questionnaire for symptom scores
p. 16 Table S4: Questionnaire for patient-reported outcome measures
p. 21 Table S5: Questionnaire for patients at *Check-Out*
p. 23 Table S6: Questionnaire for nurses at *Check-Out*
p. 25 Table S7: Variable preparation for mixed-effects models
p. 26 Table S8: Data completeness by *Evaluation* phase
p. 27 Table S9: Data throughout *Evaluation* phases stratified by relative fluid overload
p. 29 Table S10: Parameter estimates of mixed-effects models for day or phase within the project from the available-cases analysis
p. 31 Table S11: Effects of post-dialysis fluid overload and blood volume on the subsequent treatment in the overall population
p. 32 Table S12: Effects of post-dialysis fluid overload and blood volume on the subsequent treatment in the fluid overloaded population
p. 33 Table S13: Project perception of patients and nurses
p. 34 Figure S1: Long-Itch report sample
p. 35 Figure S2: Run charts
p. 36 References

Supplemental Methods 1: Estimation of pre-dialysis body composition

Body mass at time t during treatment ($M_{body,t}$, in kg) was estimated based on pre-dialysis body mass ($M_{body,pre}$, in kg) and the mass of cumulative ultrafiltration volume at time t ($M_{u,t}$, in kg), assuming that 1 L of ultrafiltered fluid is equal to 1 kg:

$$M_{body,t} \approx M_{body,pre} - M_{u,t} \quad (1)$$

Extracellular fluid volume at time t during treatment ($V_{ecf,t}$, in L), intracellular fluid volume at time t during treatment ($V_{icf,t}$, in L) and total body fluid volume at time t during treatment ($V_{tcf,t}$, in L) were computed according to Moissl et al. (1) as outlined in Equations 2-4. BMI_t is body mass index at time t during treatment (in kg/m^2), H is body height (in cm), $R_{0,t}$ is the modelled tissue resistance at zero-frequency alternating current (in Ω) and $R_{i,t}$ is the modelled intracellular tissue resistance (in Ω):

$$V_{ecf,t} = \left(\frac{0.188}{BMI_t} + 0.2883\right) \times \left(\frac{H^2 \times \sqrt{M_{body,t}}}{R_{0,t}}\right)^{\frac{2}{3}} \quad (2)$$

$$V_{icf,t} = \left(\frac{5.8758}{BMI_t} + 0.4194\right) \times \left(\frac{H^2 \times \sqrt{M_{body,t}}}{R_{i,t}}\right)^{\frac{2}{3}} \quad (3)$$

$$V_{tbf,t} = V_{ecf,t} + V_{icf,t} \quad (4)$$

Intracellular fluid volume is assumed to stay constant throughout the treatment according to previous evidence (2) $V_{icf,t}$ therefore approximates pre-dialysis intracellular fluid volume ($V_{icf,pre}$, in L):

$$V_{icf,pre} \approx V_{icf,t} \quad (5)$$

Assuming then that ultrafiltration volume at time t during treatment ($V_{u,t}$, in L) exclusively originates from the extracellular space, pre-dialysis extracellular fluid volume ($V_{ecf,pre}$, in L) and pre-dialysis total body fluid ($V_{tbf,pre}$, in L) can be calculated as:

$$V_{ecf,pre} = V_{ecf,t} + V_{u,t} \quad (6)$$

$$V_{tbf,pre} = V_{ecf,pre} + V_{icf,pre} \quad 7$$

Pre-dialysis fluid overload ($M_{fo,pre}$, in kg ≈ $V_{fo,pre}$, in L), lean tissue mass ($M_{lt,pre}$, in kg) and adipose tissue mass ($M_{at,pre}$, in kg) were calculated according to Chamney et al. (3):

$$M_{fo,pre} = 1.136 \times V_{ecf,pre} - 0.430 \times V_{icf,pre} - 0.114 \times M_{body,pre} \quad 8$$

$$V_{fo,pre} \approx M_{fo,pre} \quad 9$$

$$F_{fo/ecf,pre} = V_{fo,pre} - V_{ecf,pre} \quad 10$$

$$M_{lt,pre} = 2.725 \times V_{icf,pre} + 0.191 \times M_{fo,pre} \times 0.191 - M_{body,pre} \quad 11$$

$$M_{at,pre} = M_{body,pre} - M_{fo,pre} - M_{lt,pre} \quad 12$$

Post-dialysis fluid overload ($V_{fo,post}$, in L) and post-dialysis relative fluid overload ($F_{fo/ecf,\ post}$, in %) were calculated from the cumulative ultrafiltration volume (V_u, in L) as:

$$V_{fo,post} = V_{fo,pre} - V_u \quad 13$$

$$F_{fo/ecf,post} = V_{fo,post} - \left(V_{ecf,pre} - V_u\right) \quad 14$$

Supplemental Methods 2: Age-correction of fluid overload

The Body Composition Monitor (bFresenius Medical Care, Bad Homburg, Germany) appears to employ age-correction of fluid overload, which was not disclosed in the published equations (1,3) referenced on the product website (4). To describe more meaningful results, and to facilitate comparisons with previous studies which used the more common BCM compared to the Multiscan 5000 (Bodystat Ltd., Douglas, Isle of Man) used in this study, we developed, and age-correction of fluid overload based on a previously published dataset. Between November 2023 and January 2024, patients on maintenance hemodialysis at the Vienna Dialysis-Center (Vienna, Austria) received pre-dialysis bioimpedance spectroscopy measurements with the BCM. The retrospective analysis of this dataset was granted by the ethics committee of "Barmherzige Brüder Wien" on May 3rd, 2023.

We compared fluid overload taken directly from the BCM with fluid overload computed from resistances measured with the BCM based on published and referenced equations (1,3). Figure 1 shows the difference between the device- and formula-output depending on age, suggesting an overestimation of fluid overload of ~2.5 L at 80 years.

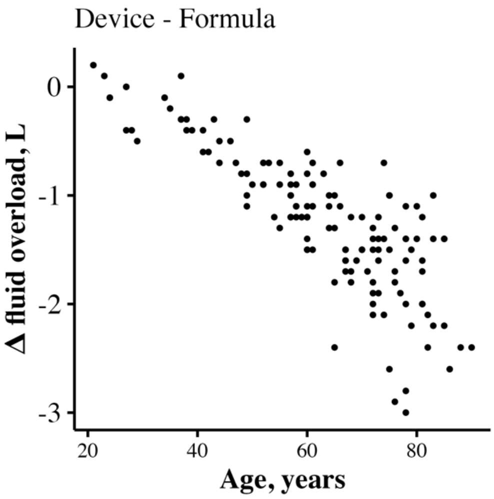

Figure 1: Difference between on-device and computed fluid overload. Δ fluid overload was computed between fluid overload taken directly from the BCM and fluid overload based on resistances taken from the BCM and computed according to published equations (1,3).

We fit a linear model with fluid overload taken from the BCM as the dependent variable, and age (in years) and calculated fluid overload (in L) as independent variables. Model estimates are listed in Table 1.

Term	Estimate	Standard Error	t-Value	P
Intercept	0.881	0.126	7.018	0.000
Fluid overload, L	0.985	0.014	72.862	0.000
Age, years	−0.033	0.002	−16.600	0.000
R-squared	0.975			
Residual Std. Error	0.372			

Table 1: Model estimates for fluid overload and age estimating on-device fluid overload from the Body Composition Monitor.

Considering the good fit of the model ($R^2 = 0.975$), we corrected all pre-dialysis fluid overload measurements of the quality improvement project according to Equation 15, where $V_{fo,pre}$ is pre-diaylsis fluid overload computed according to Equations 8 and 9.

$$V_{fo,age-corrected} = 0.881 + 0.985 \times V_{fo,pre} - 0.033 \times age \quad (15)$$

Supplemental Tables

Table S1: Questionnaire for patients at *Check-In*

Item	Question	Choices
CHIP_01	**Single Choice**<br><br>GER: Leiden Sie an Juckreiz?<br>*ENG: Do you suffer from itching?*	GER: Ja<br>*ENG: Yes*
		GER: Nein<br>*ENG: No*
CHIP_02	**Multiple Choice**<br><br>GER: Wo verspüren Sie Juckreiz?<br>*ENG: Where do you perceive itch?*	GER: Nirgends<br>*ENG: Nowhere*
		GER: Beine<br>*ENG: Legs*
		GER: Arme<br>*ENG: Arms*
		GER: Rücken<br>*ENG: Back*
		GER: Brust-& Bauchbereich<br>*ENG: Chest and abdomen*
		GER: Kopf/Nackenbereich<br>*ENG: Head or neck*
		GER: Hand- & Fußflächen<br>*ENG: Palms and foot soles*
		GER: Am ganzen Körper<br>*ENG: Across the entire body*
CHIP_03	**Single Choice**<br><br>GER: Wie würden Sie Ihren Flüssigkeitsstatus derzeit beschreiben?<br>*ENG: How would you categorize your fluid status?*	GER: Zu trocken<br>*ENG: Too dry*
		GER: Genau richtig<br>*ENG: Exactly right*
		GER: Überwässert<br>*ENG: Fluid overloaded*
CHIP_04	**Single Choice**<br><br>GER: Leiden Sie an einer durch einen Arzt/eine Ärztin festgestellten Depression?<br>*ENG: Do you suffer from depression that was diagnosed by a doctor?*	GER: Ja<br>*ENG: Yes*
		GER: Nein<br>*ENG: No*

CHIP_05	**Single Choice**<br><br>GER: Finden Sie, dass Juckreiz derzeit adäquat erhoben wird?<br>*ENG: Do you think that itch is currently being adequately assessed?*	GER: Ja<br>*ENG: Yes*
		GER: Nein<br>*ENG: No*
CHIP_06	**Single Choice**<br><br>GER: Finden Sie, dass Ihr Flüssigkeitsstatus derzeit adäquat erhoben wird?<br>*ENG: Do you think that your fluid status is currently being adequately assessed?*	GER: Ja<br>*ENG: Yes*
		GER: Nein<br>*ENG: No*
CHIP_07	**Single Choice**<br><br>GER: Finden Sie, dass depressive Symptome derzeit adäquat erhoben werden?<br>*ENG: Do you think that symptoms of depression are currently being adequately assessed?*	GER: Ja<br>*ENG: Yes*
		GER: Nein<br>*ENG: No*
CHIP_08	**Single Choice**<br><br>GER: Produzieren Sie noch Restharn?<br>*ENG: Do you have residual diuresis?*	GER: Ja<br>*ENG: Yes*
		GER: Nein<br>*ENG: No*
CHIP_09	**Numerical Input**<br><br>GER: Wieviel Restharn in Milliliter (mL) haben Sie pro Tag?<br>*ENG: How much residual diuresis in millilitres (mL) do you have per day?*	
CHIP_10	**Single Choice**<br><br>GER: Erwarten Sie, dass Sie von diesem Projekt profitieren werden?<br>*ENG: Do you expect to benefit from this project?*	GER: Ja<br>*ENG: Yes*
		GER: Nein<br>*ENG: No*

Table S2: Questionnaire for nurses at *Check-In*

Item	Question	Choices
CHIN_01	**Single Choice**<br><br>GER: Leidet der Patient / die Patientin an Juckreiz?<br>*ENG: Does this patient suffer from itching*	GER: Ja<br>*ENG: Yes*
		GER: Nein<br>*ENG: No*
CHIN_02	**Single Choice**<br><br>GER: Hat der Patient / die Patientin sichtbare Kratzspuren?<br>*ENG: Does the patient have visible scratches?*	GER: Ja<br>*ENG: Yes*
		GER: Nein<br>*ENG: No*
CHIN_03	**Multiple Choice**<br><br>GER: Bekommt der Patient / die Patientin derzeit eine medikamentöse Therapie gegen Juckreiz?<br>*ENG: Does the patient currently receive medication for itching?*	GER: Nein<br>*ENG: No*
		GER: Kapruvia®<br>*ENG: Kapruvia®*
		GER: Gabapentin<br>*ENG: Gabapentin*
		GER: Pregabalin<br>*ENG: Pregabalin*
		GER: Cortison-haltige Crème<br>*ENG: Cream containing cortisone*
		GER: Antihistaminika<br>*ENG: Antihistamines*
		GER: Anderes<br>*ENG: Other*
CHIN_04	**Single Choice**<br><br>GER: Wie würden Sie den Flüssigkeitsstatus des Patienten / der Patientin derzeit einschätzen?<br>*ENG: How would you categorize the patient's fluid status?*	GER: Zu trocken<br>*ENG: Too dry*
		GER: Genau richtig<br>*ENG: Exactly right*
		GER: Überwässert<br>*ENG: Fluid overloaded*
CHIN_05	**Single Choice**<br><br>GER: Leidet der Patient / die Patientin an einer durch einen Arzt / eine Ärztin festgestellten Depression?<br>*ENG: Does the patient suffer from depression that was diagnosed by a doctor?*	GER: Ja<br>*ENG: Yes*
		GER: Nein<br>*ENG: No*

CHIN_06	**Single Choice**<br><br>GER: Finden Sie, dass Juckreiz derzeit adäquat erhoben wird?<br>*ENG: Do you think that itch is currently being adequately assessed?*	GER: Ja<br>*ENG: Yes*
		GER: Nein<br>*ENG: No*
CHIN_07	**Single Choice**<br><br>GER: Finden Sie, dass Ihr Flüssigkeitsstatus derzeit adäquat erhoben wird?<br>*ENG: Do you think that your fluid status is currently being adequately assessed?*	GER: Ja<br>*ENG: Yes*
		GER: Nein<br>*ENG: No*
CHIN_08	**Single Choice**<br><br>GER: Finden Sie, dass depressive Symptome derzeit adäquat erhoben werden?<br>*ENG: Do you think that symptoms of depression are currently being adequately assessed?*	GER: Ja<br>*ENG: Yes*
		GER: Nein<br>*ENG: No*
CHIN_09	**Single Choice**<br><br>GER: Erwarten Sie, dass der Patient / die Patientin von diesem Projekt profitieren wird?<br>*ENG: Do you expect the patient to benefit from this project?*	GER: Ja<br>*ENG: Yes*
		GER: Nein<br>*ENG: No*
CHIN_10	**Single Choice**<br><br>GER: Erwarten Sie, dass das Pflege-Team von diesem Projekt profitieren wird?<br>*ENG: Do you exepect the nursing team to benefit from this project?*	GER: Ja<br>*ENG: Yes*
		GER: Nein<br>*ENG: No*
CHIN_11	**Single Choice**<br><br>GER: Erwarten Sie, dass das ärztliche Team von diesem Projekt profitieren wird?<br>*ENG: Do you expect the doctors to benefit from this project?*	GER: Ja<br>*ENG: Yes*
		GER: Nein<br>*ENG: No*

Table S3: Questionnaire for symptom scores

Item	Question	Choices
SYMP_01	GER: In welche dieser drei Kategorien ordnet sich der Patient / die Patientin innerhalb der letzten 14 Tage am ehesten ein?<br><br>*ENG: In which of these three categories does the patient most likely categorize himself/herself within the last 14 days?*	
SYMP_02	**Single Choice**<br><br>GER: Beispiel: Wenn der Patient / die Patientin angibt, sich regelmäßig "blutig" zu kratzen, aber keine Schlafprobleme angibt, ist er / sie in Kategorie 3 einzuordnen.<br>*ENG: Example: If the patient states that he/she regularly scratches him/herself 'bloody', but does not report any sleep problems, he/she should be categorized in category 3.*	GER: 1 - Mild: Ich habe normalerweise keine Kratzspuren auf meiner Haut. Ich habe normalerweise keine Schlafprobleme aufgrund von Juckreiz. Juckreiz löst in mir weder Wut noch Traurigkeit aus.<br><br>*ENG: 1 - Mild: I do not generally have scratch marks on my skin. I do not generally have a problem sleeping because of itching. My itching does not generally make me feel agitated or sad.*
		GER: 2 - Moderat: Ich habe manchmal Kratzspuren auf der Haut. Ich habe manchmal Schlafprobleme aufgrund von Juckreiz. Juckreiz löst in mir manchmal Wut oder Traurigkeit aus.<br><br>*ENG: 2 - Moderate: I sometimes have scratch marks on my skin. I sometimes have problems sleeping because of itching. My itching can sometimes make me feel agitated or sad.*
		GER: 3 - Schwer: Ich habe oft Kratzspuren auf der Haut, die bluten oder sich infizieren können, aber nicht müssen. Ich habe oft Schlafprobleme aufgrund von Juckreiz. Juckreiz löst in mir oft Wut oder Traurigkeit aus.<br><br>*ENG: 3 - Severe: I often have scratch marks on my skin that may or may not bleed or get infected. I often have a problem sleeping because of itching. My itching often makes me feel agitated or sad.*

SYMP_03	**Multiple Choice**<br><br>GER: Bekommt der Patient / die Patientin derzeit eine medikamentöse Therapie gegen Juckreiz?<br>*ENG: Is the patient currently receiving medication to treat itching?*	GER: Nein.<br>*ENG: No.*
		GER: Kapruvia®<br>*ENG: Kapruvia®*
		GER: Gabapentin<br>*ENG: Gabapentin*
		GER: Pregabalin<br>*ENG: Pregabalin*
		GER: Cortison-haltige Crème<br>*ENG: Cream containing cortisone*
		GER: Antihistaminika<br>*ENG: Antihistamines*
		GER: Anderes<br>*ENG: Other*
SYMP_04	GER: Wie würden Sie den Flüssigkeitsstatus des Patienten / der Patientin auf Basis folgender Fragen einschätzen?<br>*ENG: How would you assess the patient's fluid status based on the following questions?*	
SYMP_05	**Single Choice**<br><br>GER: Wie stark war in den letzten 14 Tagen die prädialytisch ausgeprägteste Atemnot des Patienten / der Patientin?<br>*ENG: How severe has the patient's pre-dialysis breathlessness been in the last 14 days?*	GER: Keine<br>*ENG: None*
		GER: Im Liegen<br>*ENG: Lying down*
		GER: Im Liegen mit zwei Kissen unter dem Kopf<br>*ENG: Lying down with two pillows under your head*
		GER: Im Sitzen<br>*ENG: Sitting*
SYMP_06	**Single Choice**<br><br>GER: Wie stark waren in den letzten 14 Tagen die prädialytisch ausgeprägtesten Knöchelödeme des Patienten / der Patientin?<br>*ENG: How severe have the patient's pre-dialysis ankle oedema been in the last 14 days?*	GER: Keine<br>*ENG: None*
		GER: Schwachausgeprägt<br>*ENG: Moderately severe*
		GER: Stark ausgeprägt<br>*ENG: Severe*

SYMP_07	**Single Choice**<br><br>GER: Traten in den letzten 14 Tagen folgende Zeichen der Volumenexpansion zwischen Dialysen auf?<br>*ENG: Have the following signs of volume expansion occurred in the last 14 days between dialyses?*	GER: Keine<br>*ENG: None*
		GER: Unerwartet geringe Zunahme der Körpermasse<br>*ENG: Unexpectedly small increase in body mass*
		GER: Chronischer, neu aufgetretener Husten<br>*ENG: Chronic, new onset cough*
SYMP_08	**Single Choice**<br><br>GER: Stieg der Blutdruck in den letzten 14 Tagen während der Dialyse eher an?<br>*ENG: Did blood pressure tend to increase during dialysis in the last 14 days?*	GER: Nein<br>*ENG: No*
		GER: Ja<br>*ENG: Yes*
SYMP_09	**Single Choice**<br><br>GER: Wie stark waren in den letzten 14 Tagen die postdialytisch ausgeprägtesten Wadenkrämpfe des Patienten / der Patientin?<br>*ENG: In the last 14 days, how severe were the patient's most pronounced postdialytic calf cramps of the patient?*	GER: Keine<br>*ENG: None*
		GER: Schwach ausgeprägt<br>*ENG: Weak*
		GER: Stark ausgeprägt<br>*ENG: Severe*
SYMP_10	**Single Choice**<br><br>GER: Kam es in den letzten 14 lagen zu symptomatischen intradialytischen Hypotensionen und einem Abfall des systolischen Blutdrucks um ≥ 20 mmHg? Wie wurde interveniert?<br>*ENG: Did symptomatic intradialytic hypotension and a drop in systolic blood pressure of ≥ 20 mmHg occur in the last 14 days? What was the reaction?*	GER: Keine<br>*ENG: None*
		GER: Wechsel der Körperposition<br>*ENG: Change of body position necessary*
		GER: Kochsalzinfusion oder Stopp der Ultrafiltration nötig<br>*ENG: Saline infusion or stop ultrafiltration necessary*
		GER: Erbrechen oder Bewusstlosigkeit als Folge<br>*ENG: Vomiting or unconsciousness as a result*
SYMP_11	**Single Choice**<br><br>GER: Wie stark war in den letzten 14 Tagen das am stärksten ausgeprägteste Zeichen von Volumendepletion, das Sie bei diesem Patienten mitbekommen haben?<br>*ENG: How strong was the most pronounced sign of volume depletion that you have seen in this patient in the last 14 days?*	GER: Keine<br>*ENG: None*
		GER: Durst direkt nach der Dialyse<br>*ENG: Thirst right after dialysis*
		GER: Schlaffheit oder Müdigkeit<br>*ENG: Sleepiness or tiredness*
		GER: Schwindel oder symptomatische Hypotension<br>*ENG: Dizziness or symptomatic hypotension*
SYMP_12	GER: Bitte fragen Sie den Patienten / die Patientin, wie oft er / sie sich im Verlauf der letzten 14 Tage durch die folgenden Beschwerden beeinträchtigt gefühlt hat:<br>*ENG: Please ask the patient how often he/she has felt affected by the following complaints in the last 14 days:*	

SYMP_13	**Single Choice**<br><br>GER: Wenig Interesse oder Freude an Ihren Tätigkeiten<br>*ENG: Little interest or pleasure in doing things*	GER: Überhaupt nicht<br>*ENG: Not at all*
		GER: An einzelnen Tagen<br>*ENG: Several days*
		GER: An mehr als der Hälfte der Tage<br>*ENG: More than half the days*
		GER: Beinahe jeden Tag<br>*ENG: Nearly every day*
SYMP_14	**Single Choice**<br><br>GER: Niedergeschlagenheit, Schwermut oder Hoffnungslosigkeit<br>*ENG: Feeling down, depressed, or hopeless*	GER: Überhaupt nicht<br>*ENG: Not at all*
		GER: An einzelnen Tagen<br>*ENG: Several days*
		GER: An mehr als der Hälfte der Tage<br>*ENG: More than half the days*
		GER: Beinahe jeden Tag<br>*ENG: Nearly every day*
SYMP_15	**Single Choice**<br><br>GER: Schwierigkeiten ein- oder durchzuschlafen oder vermehrter Schlaf<br>*ENG: Trouble falling or staying asleep, or sleeping too much*	GER: Überhaupt nicht<br>*ENG: Not at all*
		GER: An einzelnen Tagen<br>*ENG: Several days*
		GER: An mehr als der Hälfte der Tage<br>*ENG: More than half the days*
		GER: Beinahe jeden Tag<br>*ENG: Nearly every day*
SYMP_16	**Single Choice**<br><br>GER: Müdigkeit oder Gefühl, keine Energie zu haben<br>*ENG: Feeling tired or having little energy*	GER: Überhaupt nicht<br>*ENG: Not at all*
		GER: An einzelnen Tagen<br>*ENG: Several days*
		GER: An mehr als der Hälfte der Tage<br>*ENG: More than half the days*
		GER: Beinahe jeden Tag<br>*ENG: Nearly every day*

SYMP_17	**Single Choice**<br><br>GER: Verminderter Appetit oder übermäßiges Bedürfnis zu essen<br>*ENG: Poor appetite or overeating*	GER: Überhaupt nicht<br>*ENG: Not at all*<br>GER: An einzelnen Tagen<br>*ENG: Several days*<br>GER: An mehr als der Hälfte der Tage<br>*ENG: More than half the days*<br>GER: Beinahe jeden Tag<br>*ENG: Nearly every day*
SYMP_18	**Single Choice**<br><br>GER: Schlechte Meinung von sich selbst; Gefühl, ein Versager zu sein oder die Familie enttäuscht zu haben<br>*ENG: Feeling bad about yourself — or that you are a failure or have let yourself or your family down*	GER: Überhaupt nicht<br>*ENG: Not at all*<br>GER: An einzelnen Tagen<br>*ENG: Several days*<br>GER: An mehr als der Hälfte der Tage<br>*ENG: More than half the days*<br>GER: Beinahe jeden Tag<br>*ENG: Nearly every day*
SYMP_19	**Single Choice**<br><br>GER: Schwierigkeiten, sich auf etwas zu konzentrieren, z.B. beim Zeitunglesen oder Fernsehen<br>*ENG: Trouble concentrating on things, such as reading the newspaper or watching television*	GER: Überhaupt nicht<br>*ENG: Not at all*<br>GER: An einzelnen Tagen<br>*ENG: Several days*<br>GER: An mehr als der Hälfte der Tage<br>*ENG: More than half the days*<br>GER: Beinahe jeden Tag<br>*ENG: Nearly every day*
SYMP_20	**Single Choice**<br><br>GER: Waren Ihre Bewegungen oder Ihre Sprache so verlangsamt, dass es auch anderen auffallen würde? Oder waren Sie im Gegenteil „zappelig" oder ruhelos und hatten dadurch einen stärkeren Bewegungsdrang als sonst?<br>*ENG: Moving or speaking so slowly that other people could have noticed? Or the opposite — being so fidgety or restless that you have been moving around a lot more than usual*	GER: Überhaupt nicht<br>*ENG: Not at all*<br>GER: An einzelnen Tagen<br>*ENG: Several days*<br>GER: An mehr als der Hälfte der Tage<br>*ENG: More than half the days*<br>GER: Beinahe jeden Tag<br>*ENG: Nearly every day*

SYMP_21	**Single Choice**<br><br>GER: Gedanken, dass Sie lieber tot wären oder sich Leid zufügen möchten<br>*ENG: Thoughts that you would be better off dead or of hurting yourself in some way*	GER: Überhaupt nicht<br>*ENG: Not at all*
		GER: An einzelnen Tagen<br>*ENG: Several days*
		GER: An mehr als der Hälfte der Tage<br>*ENG: More than half the days*
		GER: Beinahe jeden Tag<br>*ENG: Nearly every day*

Table S4: Questionnaire for patient-reported outcome measures

Item	Question	Choices
PROM_01	**Single Choice**<br><br>*GER: Wie fühlten Sie sich heute direkt nach Ihrer Dialyse?*<br>*ENG: How did you feel right after dialysis today?*	GER: 😃 Sehr gut<br>*ENG:* 😃 Very good
		GER: 😌 Good<br>*ENG:* 😌 *Good*
		GER: 😐 Neutral<br>*ENG:* 😐 *Neutral*
		GER: 😕 Eher schlecht<br>*ENG:* 😕 *Rather bad*
		GER: 😣 Schlecht<br>*ENG:* 😣 *Bad*
PROM_02	**Single Choice**<br><br>GER: Wählen Sie jene Zahl aus, welche die Qualität Ihres Schlafes in den letzten 24 Stunden am besten beschreibt!<br>*ENG: Choose the number best representing your sleep quality within the previous 24 hours!*	GER: 0 – Bestmöglicher Schlaf<br>*ENG: 0 – Best possible sleep*
		1
		2
		3
		4
		5
		6
		7
		8
		9
		GER: 10 – Schlechtester Schlaf<br>*ENG: 10 – Worst sleep*

PROM_03	**Single Choice**<br><br>GER: Bitte bewerten Sie den schlimmsten Juckreiz, den Sie den letzten 24 Stunden verspürt haben!<br>*ENG: Please rate the worst itch you have perceived during the previous 24 hours!*	GER: 0 – Kein Juckreiz<br>*ENG: 0 – No tich*
		GER: 1 – Mild<br>*ENG: 1 – Mild*
		GER: 2 – Mild<br>*ENG: 2 – Mild*
		GER: 3 – Mild<br>*ENG: 3 – Mild*
		GER: 4 – Moderat<br>*ENG: 4 – Moderat*
		GER: 5 – Moderat<br>*ENG: 5 – Moderat*
		GER: 6 – Moderat<br>*ENG: 6 – Moderat*
		GER: 7 – Stark<br>*ENG: 7 – Severe*
		GER: 8 – Stark<br>*ENG: 8 – Severe*
		GER: 9 – Schlimmster vorstellbarer Juckreiz<br>*ENG: 9 – Worst imaginable itch*
		GER: 10 – Schlimmster vorstellbarer Juckreiz<br>*ENG: 10 – Worst imaginable itch*
PROM_04	**Single Choice**<br><br>GER: Wie lange hat es nach der letzten Dialyse gedauert, bis Sie sich erholt haben?<br>*ENG: When did you recover after the previous dialysis treatment?*	GER: Weniger als 2 Stunden<br>*ENG: Less than 2 hours*
		GER: 2 bis 6 Stunden<br>*ENG: 2 to 6 hours*
		GER: 7 bis 12 Stunden<br>*ENG: 7 to 12 hours*
		GER: Mehr als 12 Stunden<br>*ENG: More than 12 hours*

PROM_05	**Single Choice**<br><br>GER: Wie schätzen Sie Ihre heutige Dialyse ein?<br>*ENG: How would you rate today's dialysis?*	GER: 😃 Sehr gut<br>*ENG:* 😃 Very good<br>GER: 😌 Good<br>*ENG:* 😌 *Good*<br>GER: 😐 Neutral<br>*ENG:* 😐 *Neutral*<br>GER: 😕 Eher schlecht<br>*ENG:* 😕 *Rather bad*<br>GER: 😣 Schlecht<br>*ENG:* 😣 *Bad*
PROM_06	**Multiple Choice**<br><br>GER: Gab es besondere Vorkommnisse während der heutigen Dialyse?<br>*ENG: Did any complications occur during today's dialysis?*	GER: Nein<br>*ENG: No*<br>GER: Spürbarer Abfall des Blutdrucks<br>*ENG: Perceivable drop in blood pressure*<br>GER: Spürbarer Anstieg des Blutdrucks<br>*ENG: Perceivable rise in blood pressure*<br>GER: Blutung<br>*ENG: Bleeding*<br>GER: Probleme mit dem Dialysezugang<br>*ENG: Dialysis access issues*<br>GER: Krämpfe<br>*ENG: Cramps*
PROM_07	**Multiple Choice**<br><br>GER: Was wurde gegen den Blutdruckabfall unternommen?<br>*ENG: Which measure was taken to mitigate the drop in blood pressure?*	GER: Nichts<br>*ENG: None*<br>GER: Änderung der Körperposition<br>*ENG: Change In body position*<br>GER: Infusion von Flüssigkeit<br>*ENG: Fluid infusion*
PROM_08	**Numerical Input**<br><br>GER: Wie hoch war Ihr Körpergewicht nach der heutigen Dialyse?<br>*ENG: What was your body weight after dialysis today?*	

PROM_09	**Numerical Input**<br><br>GER: Wie hoch war Ihr heutiges Zielgewicht?<br>*ENG: What was your target weight today?*	
PROM_10	**Single Choice**<br><br>GER: Ziehen Sie von Ihrem Körpergewicht etwas für Ihre Kleidung ab?<br>*ENG: Do you deduct weight from your body weight to account for clothing?*	GER: Ja<br>*ENG: Yes*
		GER: Nein<br>*ENG: No*
PROM_11	**Multiple Choice**<br><br>GER: Bekommen Sie derzeit eine medikamentöse Therapie gegen Juckreiz?<br>*ENG: Do you currently receive medication for itching?*	GER: Nein<br>*ENG: No*
		GER: Kapruvia®<br>*ENG: Kapruvia®*
		GER: Gabapentin<br>*ENG: Gabapentin*
		GER: Pregabalin<br>*ENG: Pregabalin*
		GER: Cortison-haltige Crème<br>*ENG: Cream containing cortisone*
		GER: Antihistaminika<br>*ENG: Antihistamines*
		GER: Anderes<br>*ENG: Other*
PROM_12	**Multiple Choice**<br><br>GER: Wurde seit der letzten Dialyse eine Änderung Ihres Trockengewichts durchgeführt?<br>*ENG: Was your dry weight changed since the last dialysis treatment?*	GER: Nein<br>*ENG: No*
		GER: Ja, von mir<br>*ENG: Yes, by myself*
		GER: Ja, von der Pflegeperson<br>*ENG: Yes, by the nurse*
		GER: Ja, von der Ärzteschaft<br>*ENG: Yes, by the doctors*

PROM_13	**Multiple Choice**<br><br>GER: Wurde Ihnen seit der letzten Dialyse zu einer psychaitrischen Begutachtung geraten?<br>*ENG: Did you receive a recommendation to see a psychiatrist?*	GER: Nein<br>*ENG: No*
		GER: Ja, von der Pflegeperson<br>*ENG: Yes, by the nurse*
		GER: Ja, von der Ärzteschaft<br>*ENG: Yes, by the doctors*

Table S5: Questionnaire for patients at *Check-Out*

Item	Question	Choices
CHOP_01	**Single Choice**<br><br>GER: Leiden Sie an Juckreiz?<br>*ENG: Do you suffer from itching?*	GER: Ja<br>*ENG: Yes*
		GER: Nein<br>*ENG: No*
CHOP_02	**Multiple Choice**<br><br>GER: Wo verspüren Sie Juckreiz?<br>*ENG: Where do you perceive itch?*	GER: Nirgends<br>*ENG: Nowhere*
		GER: Beine<br>*ENG: Legs*
		GER: Arme<br>*ENG: Arms*
		GER: Rücken<br>*ENG: Back*
		GER: Brust-& Bauchbereich<br>*ENG: Chest and abdomen*
		GER: Kopf/Nackenbereich<br>*ENG: Head or neck*
		GER: Hand- & Fußflächen<br>*ENG: Palms and foot soles*
		GER: Am ganzen Körper<br>*ENG: Across the entire body*
CHOP_03	**Single Choice**<br><br>GER: Wie würden Sie Ihren Flüssigkeitsstatus derzeit beschreiben?<br>*ENG: How would you categorize your fluid status?*	GER: Zu trocken<br>*ENG: Too dry*
		GER: Genau richtig<br>*ENG: Exactly right*
		GER: Überwässert<br>*ENG: Fluid overloaded*
CHOP_04	**Single Choice**<br><br>GER: Leiden Sie an einer durch einen Arzt/eine Ärztin festgestellten Depression?<br>*ENG: Do you suffer from depression that was diagnosed by a doctor?*	GER: Ja<br>*ENG: Yes*
		GER: Nein<br>*ENG: No*

CHOP_05	**Single Choice**<br><br>GER: Finden Sie, dass im Rahmen des Projekts Juckreiz adäquat erhoben wurde?<br>*ENG: Do you think that itch was adequately assessed during the project?*	GER: Ja<br>*ENG: Yes*
		GER: Nein<br>*ENG: No*
CHOP_06	**Single Choice**<br><br>GER: War Ihnen der Aufwand zur Juckreiz-Erhebung zu hoch?<br>*ENG: Did itch assessment require too much effort?*	GER: Ja<br>*ENG: Yes*
		GER: Nein<br>*ENG: No*
CHOP_07	**Single Choice**<br><br>GER: Finden Sie, dass im Rahmen des Projekts Ihr Flüssigkeitsstatus erhoben wurde?<br>*ENG: Do you think that your fluid status was adequately assessed during the project?*	GER: Ja<br>*ENG: Yes*
		GER: Nein<br>*ENG: No*
CHOP_08	**Single Choice**<br><br>GER: War Ihnen der Aufwand zur Flüssigkeits-Erhebung zu hoch?<br>*ENG: Did fluid status assessment require too much effort?*	GER: Ja<br>*ENG: Yes*
		GER: Nein<br>*ENG: No*
CHOP_09	**Single Choice**<br><br>GER: Finden Sie, dass im Rahmen des Projekts depressive Symptome adäquat erhoben wurden?<br>*ENG: Do you think that symptoms of depression were adequately assessed during the project?*	GER: Ja<br>*ENG: Yes*
		GER: Nein<br>*ENG: No*
CHOP_10	**Single Choice**<br><br>GER: War Ihnen der Aufwand zur Depressions-Erhebung zu hoch?<br>*ENG: Did depression assessment require too much effort?*	GER: Ja<br>*ENG: Yes*
		GER: Nein<br>*ENG: No*
CHOP_11	**Single Choice**<br><br>GER: Finden Sie, dass Sie von diesem Projekt insgesamt profitiert haben?<br>*ENG: Do you believe to have benefited from this project overall?*	GER: Ja<br>*ENG: Yes*
		GER: Nein<br>*ENG: No*

Table S6: Questionnaire for nurses at *Check-Out*

Item	Question	Choices
CHON_01	**Single Choice**<br><br>GER: Leidet der Patient / die Patientin an Juckreiz?<br>*ENG: Does this patient suffer from itching*	GER: Ja<br>*ENG: Yes*
		GER: Nein<br>*ENG: No*
CHON_02	**Single Choice**<br><br>GER: Hat der Patient / die Patientin sichtbare Kratzspuren?<br>*ENG: Does the patient have visible scratches?*	GER: Ja<br>*ENG: Yes*
		GER: Nein<br>*ENG: No*
CHON_03	**Multiple Choice**<br><br>GER: Bekommt der Patient / die Patientin derzeit eine medikamentöse Therapie gegen Juckreiz?<br>*ENG: Does the patient currently receive medication for itching?*	GER: Nein<br>*ENG: No*
		GER: Kapruvia®<br>*ENG: Kapruvia®*
		GER: Gabapentin<br>*ENG: Gabapentin*
		GER: Pregabalin<br>*ENG: Pregabalin*
		GER: Cortison-haltige Crème<br>*ENG: Cream containing cortisone*
		GER: Antihistaminika<br>*ENG: Antihistamines*
		GER: Anderes<br>*ENG: Other*
CHON_04	**Single Choice**<br><br>GER: Wie würden Sie den Flüssigkeitsstatus des Patienten / der Patientin derzeit einschätzen?<br>*ENG: How would you categorize the patient's fluid status?*	GER: Zu trocken<br>*ENG: Too dry*
		GER: Genau richtig<br>*ENG: Exactly right*
		GER: Überwässert<br>*ENG: Fluid overloaded*
CHON_05	**Single Choice**<br><br>GER: Leidet der Patient / die Patientin an einer durch einen Arzt / eine Ärztin festgestellten Depression?<br>*ENG: Does the patient suffer from depression that was diagnosed by a doctor?*	GER: Ja<br>*ENG: Yes*
		GER: Nein<br>*ENG: No*

CHON_06	**Single Choice**<br><br>GER: Finden Sie, dass im Rahmen des Projekts bei diesem Patienten / dieser Patientin Juckreiz adäquat erhoben wurde?<br>*ENG: Do you think that itch was adequately assessed in this patient during the project?*	GER: Ja<br>*ENG: Yes*
		GER: Nein<br>*ENG: No*
CHON_07	**Single Choice**<br><br>GER: Finden Sie, dass im Rahmen des Projekts bei diesem Patienten / dieser Patientin der Flüssigkeitsstatus adäquat erhoben wurde?<br>*ENG: Do you think that the fluid status was adequately assessed in this patient during the project?*	GER: Ja<br>*ENG: Yes*
		GER: Nein<br>*ENG: No*
CHON_08	**Single Choice**<br><br>GER: Finden Sie, dass im Rahmen des Projekts bei diesem Patienten / dieser Patientin Depression adäquat erhoben wurde?<br>*ENG: Do you think depression was adequately assessed in this patient during the project?*	GER: Ja<br>*ENG: Yes*
		GER: Nein<br>*ENG: No*

Table S7: Variable preparation for mixed-effects models

DV	IVOI	Preparation
Pre-dialysis body mass	Phase (1-3)	Mean per phase
Post-dialysis body mass	Phase (1-3)	Mean per phase
Target body mass	Phase (1-3)	Mean per phase
Euvolemic body mass	Phase (1-3)	Mean per phase
Lean tissue mass	Phase (1-3)	Mean per phase
Adipose tissue mass	Phase (1-3)	Mean per phase
Target body mass in euvolemic range	Phase	Last per phase
Post-dialysis body mass in euvolemic range	Day	None
Δ target - euvolemic body mass	Phase (1-3)	Mean per phase
Δ post-dialysis - euvolemic body mass	Phase (1-3)	Mean per phase
Pre-dialysis fluid overload	Phase (1-3)	Mean per phase
Pre-dialysis relative fluid overload	Phase (1-3)	Mean per phase
Relative fluid overload >15%	Day	None
Post-dialysis fluid overload	Phase (1-3)	Mean per phase
Post-dialysis relative fluid overload	Phase (1-3)	Mean per phase
Pre-dialysis blood volume	Phase (1-3)	Mean per phase
Pre-dialysis systolic blood pressure	Phase (1-3)	Mean per phase
Pre-dialysis diastolic blood pressure	Phase (1-3)	Mean per phase
Intradialytic hypotension	Day	None
Number of antihypertensive medications	Phase (1-3)	Last per phase
RECOVA symptom score	Phase (1-3)	Last per phase
Recovery time	Phase (1-3)	Mode per phase
Dialysis satisfaction	Phase (1-3)	Mode per phase
Well-being	Phase (1-3)	Mode per phase
Symptomatic hypotension or cramps	Day	None

Legend to Table S7: Abbreviations: DV, dependent variable; IVOI, independent variable of interest.

Table S8: Data completeness by *Evaluation* phase

Variable	Evaluation 1<br>N = 123	Evaluation 2<br>N = 121	Evaluation 3<br>N = 117
Number of documented treatments per patient	6 (6, 6)	9 (8, 9)	6 (6, 6)
Completeness of documented treatments, %	100 (100, 100)	100 (89, 100)	100 (100, 100)
At least one documented treatment per patient	122 (99%)	120 (99%)	116 (99%)
Number of blood volume measurements per patient	3 (1, 4)	3 (1, 5)	2 (0, 4)
Completeness of blood volume measurements, %	50 (17, 67)	33 (11, 56)	33 (0, 67)
At least one blood volume measurement per patient	98 (80%)	92 (76%)	71 (61%)
Number of bioimpedance spectroscopy measurements per patient	3 (2, 4)	3 (2, 4)	3 (2, 4)
Completeness of bioimpedance spectroscopy measurements, %	50 (33, 67)	33 (22, 44)	50 (33, 67)
At least one bioimpedance spectroscopy measurement per patient	111 (90%)	111 (92%)	102 (87%)
At least one symptom score per patient	110 (89%)	62 (51%)	106 (91%)
Number of PROM per patient	3 (1, 4)	2 (1, 3)	1 (0, 2)
Completeness of PROM, %	50 (17, 67)	22 (11, 33)	17 (0, 33)
At least one PROM per patient	106 (86%)	97 (80%)	69 (59%)

Legend to Table S8: The data are reported as frequency (percentage) or median (quartile 1, quartile 3) stratified by *Evaluation* phase. The number below indicates the total number of patients who provided data during the respective phase. Abbreviations: PROM, patient-reported outcome measure.

Table S9: Data throughout *Evaluation* phases stratified by relative fluid overload

Characteristic	<7%			7-15%			>15%		
	Evaluation 1 N = 99	Evaluation 2 N = 126	Evaluation 3 N = 87	Evaluation 1 N = 281	Evaluation 2 N = 385	Evaluation 3 N = 283	Evaluation 1 N = 264	Evaluation 2 N = 384	Evaluation 3 N = 272
**Body composition**									
Pre-dialysis body mass, kg	74.18 (18.80)	74.20 (18.78)	72.41 (19.49)	85.02 (19.47)	84.93 (19.73)	84.53 (19.61)	76.90 (16.12)	76.34 (16.12)	75.72 (16.11)
Post-dialysis body mass, kg	72.79 (18.36)	73.08 (18.49)	71.34 (19.07)	83.01 (18.89)	82.91 (19.16)	82.61 (19.05)	74.84 (16.07)	74.14 (15.95)	73.48 (15.85)
Target body mass, kg	72.26 (18.30)	72.70 (18.63)	71.22 (19.13)	82.50 (18.74)	82.53 (19.07)	82.29 (18.98)	74.41 (16.21)	73.80 (16.01)	73.36 (15.90)
Euvolemic body mass, kg	73.66 (18.54)	73.05 (19.73)	69.63 (19.53)	82.78 (19.04)	83.03 (19.58)	82.34 (20.07)	72.57 (15.56)	72.34 (16.02)	73.06 (16.36)
Lean tissue mass, kg	43.67 (15.36)	42.59 (15.58)	41.44 (16.86)	40.31 (9.82)	41.94 (9.79)	43.18 (11.24)	37.77 (9.08)	39.21 (9.60)	38.90 (10.64)
Adipose tissue mass, kg	29.99 (17.49)	30.46 (18.87)	28.20 (21.30)	42.47 (16.86)	41.09 (18.11)	39.16 (16.90)	34.80 (15.52)	33.13 (16.14)	34.16 (17.88)
Target body mass in euvolemic range	39 (42%)	24 (21%)	20 (31%)	203 (75%)	264 (71%)	155 (66%)	85 (33%)	150 (41%)	95 (41%)
Post-dialysis body mass in euvolemic range	32 (34%)	28 (25%)	23 (36%)	204 (76%)	263 (70%)	162 (69%)	73 (29%)	130 (36%)	109 (47%)
Δ target - euvolemic body mass, kg	1.72 (1.37)	2.07 (1.35)	1.51 (1.03)	0.99 (0.82)	1.18 (1.15)	1.22 (1.21)	2.12 (1.38)	1.73 (1.19)	1.64 (1.15)
Δ post-dialysis - euvolemic body mass, kg	1.55 (1.09)	1.92 (1.23)	1.37 (0.91)	0.94 (0.61)	1.04 (0.85)	1.11 (1.03)	2.34 (1.33)	1.86 (1.18)	1.69 (1.07)
Body mass index, kg/m squared	25.19 (5.45)	25.19 (5.40)	24.76 (5.71)	28.88 (5.62)	28.84 (5.69)	28.59 (5.64)	26.06 (4.39)	25.83 (4.37)	25.67 (4.33)
**Fluid volumes**									
Target ultrafiltration volume, L	2.51 (0.80)	2.21 (0.94)	1.81 (1.24)	2.51 (0.67)	2.52 (0.79)	2.48 (0.82)	2.74 (0.93)	2.66 (0.68)	2.65 (0.82)
Cumulative ultrafiltration volume, L	2.07 (1.48)	1.98 (1.65)	1.70 (1.35)	2.60 (1.12)	2.63 (1.12)	2.51 (1.12)	2.67 (1.14)	2.81 (1.04)	2.83 (0.95)
Pre-dialysis fluid overload (age-adjusted), L	0.44 (0.88)	0.87 (1.34)	0.89 (1.14)	2.21 (0.65)	1.86 (0.98)	1.93 (1.42)	4.40 (1.42)	3.69 (1.46)	3.39 (1.60)
Pre-dialysis relative fluid overload (age-adjusted), %	2.5 (4.4)	4.4 (6.1)	4.5 (6.2)	11.17 (2.07)	9.41 (4.47)	9.75 (6.18)	21.5 (4.7)	18.3 (5.6)	16.9 (6.7)
Post-dialysis fluid overload (age-adjusted), L	-1.68 (1.63)	-1.01 (2.31)	-1.14 (1.60)	-0.38 (1.09)	-0.75 (1.37)	-0.52 (1.57)	1.72 (1.49)	0.92 (1.47)	0.57 (1.49)
Post-dialysis relative fluid overload (age-adjusted), %	-12 (11)	-9 (14)	-10 (10)	-3 (7)	-5 (9)	-3 (9)	9 (8)	4 (10)	2 (9)
Pre-dialysis blood volume, L	5.07 (1.32)	5.34 (1.18)	4.70 (1.12)	5.23 (1.11)	5.10 (0.89)	5.03 (1.09)	5.21 (0.93)	5.23 (1.17)	5.20 (0.92)
Post-dialysis blood volume, L	4.69 (1.36)	5.08 (1.20)	4.47 (1.22)	4.81 (1.00)	4.69 (0.85)	4.64 (1.01)	4.81 (0.93)	4.80 (1.16)	4.76 (0.88)
Pre-dialysis specific blood volume, mL/kg	71 (17)	73 (14)	71 (14)	64 (13)	64 (11)	64 (12)	71 (15)	71 (13)	69 (11)
Post-dialysis specific blood volume, mL/kg	65 (16)	70 (14)	67 (14)	59 (14)	59 (13)	59 (13)	65 (14)	65 (13)	63 (10)
**Blood pressure**									
Pre-dialysis systolic blood pressure, mmHg	125 (17)	125 (18)	121 (22)	134 (22)	132 (19)	130 (20)	139 (18)	132 (19)	131 (19)
Pre-dialysis diastolic blood pressure, mmHg	69 (10)	68 (10)	66 (12)	73 (11)	72 (11)	70 (12)	73 (13)	70 (13)	68 (10)

Characteristic	<7%			7-15%			>15%		
	**Evaluation 1 N = 99**	**Evaluation 2 N = 126**	**Evaluation 3 N = 87**	**Evaluation 1 N = 281**	**Evaluation 2 N = 385**	**Evaluation 3 N = 283**	**Evaluation 1 N = 264**	**Evaluation 2 N = 384**	**Evaluation 3 N = 272**
Intradialytic hypotension (Nadir 90/100)	6 (6.6%)	5 (4.5%)	6 (9.2%)	12 (4.6%)	36 (12%)	26 (12%)	16 (6.7%)	32 (9.8%)	23 (12%)
Symptomatic intradialytic hypotension	6 (11%)	1 (3.4%)	1 (14%)	7 (5.6%)	6 (5.1%)	3 (4.8%)	4 (3.1%)	9 (7.5%)	7 (13%)
Symptomatic intradialytic hypertension				1 (0.8%)	0 (0%)	0 (0%)	5 (3.8%)	3 (2.5%)	2 (3.8%)
**Patient-reported outcomes**									
Muscle cramps	4 (7.5%)	1 (3.4%)	1 (14%)	6 (4.8%)	7 (6.0%)	4 (6.3%)	5 (3.8%)	3 (2.5%)	1 (1.9%)
RECOVA symptom score									
0, Evaluate target mass in 2 weeks	5 (33%)	5 (63%)	2 (18%)	20 (43%)	10 (38%)	10 (23%)	13 (32%)	10 (43%)	14 (35%)
1-4, Question target mass	4 (27%)	2 (25%)	6 (55%)	16 (35%)	8 (31%)	16 (36%)	17 (41%)	7 (30%)	12 (30%)
5-6, Adjust target mass	4 (27%)	1 (13%)	2 (18%)	4 (8.7%)	6 (23%)	11 (25%)	7 (17%)	4 (17%)	9 (23%)
7+, Immediate adjustment of target mass required	2 (13%)	0 (0%)	1 (9.1%)	6 (13%)	2 (7.7%)	7 (16%)	4 (9.8%)	2 (8.7%)	5 (13%)
Number of antihypertensive medications									
0	11 (69%)	10 (63%)	7 (50%)	26 (57%)	24 (55%)	21 (47%)	24 (50%)	21 (45%)	17 (38%)
1	4 (25%)	5 (31%)	5 (36%)	15 (33%)	14 (32%)	15 (33%)	12 (25%)	10 (21%)	12 (27%)
3	0 (0%)	0 (0%)	1 (7.1%)	1 (2.2%)	1 (2.3%)	2 (4.4%)	6 (13%)	7 (15%)	7 (16%)
4 or more	1 (6.3%)	1 (6.3%)	1 (7.1%)	2 (4.3%)	3 (6.8%)	3 (6.7%)	4 (8.3%)	5 (11%)	5 (11%)
2				2 (4.3%)	2 (4.5%)	4 (8.9%)	2 (4.2%)	4 (8.5%)	4 (8.9%)
Dialysis recovery time									
0-2 hours	7 (47%)	6 (46%)	2 (40%)	22 (54%)	15 (41%)	13 (41%)	20 (48%)	19 (50%)	8 (30%)
3-6 hours	6 (40%)	5 (38%)	1 (20%)	13 (32%)	17 (46%)	10 (31%)	16 (38%)	9 (24%)	9 (33%)
7-12 hours	0 (0%)	1 (7.7%)	0 (0%)	2 (4.9%)	1 (2.7%)	2 (6.3%)	2 (4.8%)	8 (21%)	4 (15%)
more than 12 hours	2 (13%)	1 (7.7%)	2 (40%)	4 (9.8%)	4 (11%)	7 (22%)	4 (9.5%)	2 (5.3%)	6 (22%)

Legend to Table S9: The data are reported as means of patient means (standard deviation of patient means) or frequency (percentage) of available data and are stratified by *Evaluation* phase and by average relative fluid overload during *Evaluation 1* (<7%, 7-15%, >15%). Specific blood volume was normalized to post-dialysis body mass. The last number of antihypertensive medications per patient and phase, and the mode recovery time per phase were selected for reporting. Not all data were available from all dialysis treatments, hence the number of observations per variable are lower than the total number of all studied treatments (N).

Table S10: Parameter estimates of mixed-effects models for day or phase within the project from the available-cases analysis

DV	IVOI	β_{ivoi} [95% CI]	P_{ivoi}	σ_0	σ_1	N	Patients	R^2
**Body composition**								
Pre-dialysis body mass	Phase	-0.26 [-0.38, -0.15]	<0.001	15.83	0.55	342	117	1.00
Post-dialysis body mass	Phase	-0.26 [-0.37, -0.15]	<0.001	15.53	0.53	340	116	1.00
Target body mass	Phase	-0.2 [-0.29, -0.1]	0.001	15.40	0.47	340	116	1.00
Euvolemic body mass	Phase	-0.1 [-0.22, 0.02]	0.26	16.02	0.57	307	112	1.00
Lean tissue mass	Phase	0.47 [0.19, 0.76]	0.007	8.16	0.90	307	112	0.92
Adipose tissue mass	Phase	-0.58 [-0.84, -0.32]	<0.001	15.83	0.69	307	112	0.97
Target body mass in euvolemic range*	Phase	0.98 [0.83, 1.17]	0.92	6.54	NA	307	112	0.52
Post-dialysis body mass in euvolemic range*	Day	1.02 [1, 1.03]	0.14	199.36	1.08	2025	112	0.81
Δ target - euvolemic body mass	Phase	-0.03 [-0.1, 0.04]	0.58	1.51	0.26	307	112	0.73
Δ post-dialysis - euvolemic body mass	Phase	-0.06 [-0.13, 0]	0.18	1.53	0.26	307	112	0.77
**Fluid volumes**								
Pre-dialysis fluid overload	Phase	-0.13 [-0.21, -0.06]	0.003	1.94	0.30	307	112	0.90
Pre-dialysis relative fluid overload	Phase	-0.61 [-0.91, -0.31]	0.001	7.80	1.14	307	112	0.89
Relative fluid overload >15%*	Phase	0.68 [0.51, 0.91]	0.03	127.64	NA	307	112	0.90
Post-dialysis fluid overload	Phase	-0.12 [-0.2, -0.05]	0.009	2.17	0.31	307	112	0.88
Post-dialysis relative fluid overload	Phase	-0.67 [-1.1, -0.24]	0.01	12.67	1.66	307	112	0.87
Pre-dialysis blood volume	Phase	-0.03 [-0.08, 0.01]	0.37	0.88	0.03	249	99	0.74
**Blood pressure**								
Pre-dialysis systolic blood pressure	Phase	-1.49 [-2.24, -0.73]	0.001	19.92	2.72	340	116	0.82
Pre-dialysis diastolic blood pressure	Phase	-0.84 [-1.19, -0.48]	<0.001	8.62	NA	340	116	0.79
Intradialytic hypotension*	Day	1.01 [0.99, 1.02]	0.62	4.44	1.03	1916	115	0.41

DV	IVOI	β_{ivoi} [95% CI]	P_{ivoi}	σ_0	σ_1	N	Patients	R^2
Number of antihypertensive medications*	Phase	1.32 [1.1, 1.59]	0.01	1509.10	NA	343	117	0.95
**Patient-reported outcomes**								
RECOVA symptom score*	Phase	1.08 [0.95, 1.23]	0.42	2.62	1.02	266	114	0.25
Recovery time*	Phase	1.35 [1.1, 1.66]	0.02	4.07	1.16	263	113	0.59
Dialysis satisfaction*	Phase	0.92 [0.74, 1.13]	0.59	7.50	NA	262	113	0.57
Well-being*	Phase	0.88 [0.52, 1.52]	0.81	3232107.35	39.23	265	113	0.96
Symptomatic hypotension or cramps*	Day	1 [1, 1]	0.89	7.84	1.03	733	113	0.52

Legend to Table S10: Data from patients who had at least one observation of the dependent variable (DV) regardless of the completeness across *Evaluation* phases were included in this analysis. DV were modelled with generalized linear mixed-effects models, with either project day or *Evaluation* phase as the independent variable of interest (IVOI). Models included baseline age (z-score), sex, type 2 diabetes mellitus, heart failure, albumin concentration (z-score), hemoglobin concentration (z-score) and dialysis vintage (z-score) as covariates, "β_{ivoi} [95% CI]" denotes the fixed effect estimate of the IVOI. Model parameters of DVs marked with an asterisk (*) were reported as odds ratios, and as regression coefficients otherwise. σ_0 and σ_1 indicate the standard deviation of intercepts and slopes between patients. If σ_1 is NA, the model was fit without random slopes. Abbreviations: CI, confidence interval; DV, dependent variable; IVOI, independent variable of interest; SD, standard deviation.

Table S11: Effects of post-dialysis fluid overload and blood volume on the subsequent treatment in the overall population

DV	IVOI	β_{ivoi} [95% CI]	P_{ivoi}	$P_{ivoi:delay}$	σ_0	σ_1	N	Patients	R^2
RECOVA symptom score*	Post-dialysis fluid overload	0.74 [0.21, 2.62]	0.81	0.98	1.86	1.42	95	65	0.36
RECOVA symptom score*	Post-dialysis blood volume	1.02 [0.5, 2.08]	0.97	0.57	5.47	1.12	100	61	0.38
Pre-dialysis blood volume	Post-dialysis fluid overload	0.03 [-0.21, 0.27]	0.90	0.94	0.86	NA	340	84	0.66
Pre-dialysis blood volume	Post-dialysis blood volume	0.59 [0.46, 0.71]	<0.001	0.05	0.40	NA	457	86	0.49
Symptomatic hypotension or cramps*	Post-dialysis fluid overload	0.74 [0.22, 2.49]	0.81	0.92	3.43	NA	289	96	0.36
Symptomatic hypotension or cramps*	Post-dialysis blood volume	1.65 [0.8, 3.42]	0.36	0.48	4.20	NA	265	82	0.43
Dialysis satisfaction*	Post-dialysis fluid overload	0.91 [0.4, 2.05]	0.90	0.89	3.40	1.21	282	94	0.36
Dialysis satisfaction*	Post-dialysis blood volume	0.97 [0.56, 1.7]	0.94	0.91	61.58	2.24	256	80	0.51
Recovery time*	Post-dialysis fluid overload	1.45 [0.66, 3.18]	0.57	0.50	5.46	1.50	285	95	0.56
Recovery time*	Post-dialysis blood volume	1.57 [0.96, 2.57]	0.18	0.02	1.36	1.46	259	81	0.65
Pre-dialysis fluid overload	Post-dialysis fluid overload	0.43 [0.00, 0.87]	0.15	0.93	0.67	0.27	404	105	0.57
Pre-dialysis fluid overload	Post-dialysis blood volume	-0.16 [-0.43, 0.12]	0.47	0.18	2.46	0.39	335	83	0.70
Hypertension medications*	Post-dialysis fluid overload	1.62 [0.98, 2.65]	0.15	0.26	13407.19	NA	897	112	0.97
Hypertension medications*	Post-dialysis blood volume	0.78 [0.55, 1.1]	0.32	0.56	11623.97	NA	870	97	0.97
Intradialytic hypotension*	Post-dialysis fluid overload	0.83 [0.4, 1.7]	0.79	0.92	6.10	NA	818	117	0.50
Intradialytic hypotension*	Post-dialysis blood volume	0.87 [0.56, 1.35]	0.72	0.76	4.90	NA	841	102	0.44
Well-being*	Post-dialysis fluid overload	0.72 [0.35, 1.51]	0.59	0.59	3.83	NA	286	96	0.41
Well-being*	Post-dialysis blood volume	0.98 [0.63, 1.51]	0.94	0.94	4.00	NA	262	82	0.44

Legend to Table S11: Data from all phases and patients were analyzed. Dependent variables (DV) were modelled with generalized linear mixed-effects models. Post-dialysis fluid overload and blood volume were fit as independent variables of interest (IVOI) delayed by 2-3 days in relation to the DV, with baseline age (z-score), sex, type 2 diabetes mellitus, heart failure, albumin concentration (z-score), hemoglobin concentration (z-score) and dialysis vintage (z-score) as additional fixed effects, and an interaction term between the IVOI and the time delay. "β_{ivoi} [95% CI]" denotes the effect estimates of the IVOI. Estimates marked with an asterisk (*) were reported as odds ratios, and as regression coefficients otherwise. σ_0 and σ_1 indicate the standard deviation of intercepts and slopes between patients. If σ_1 is NA, the model was fit without random slopes. Abbreviations: CI, confidence interval; DV, dependent variable; IVOI, independent variable of interest; SD, standard deviation.

Table S12: Effects of post-dialysis fluid overload and blood volume on the subsequent treatment in the fluid overloaded population

DV	IVOI	β_{ivoi} [95% CI]	P_{ivoi}	$P_{ivoi:delay}$	σ_0	σ_1	N	Patients	R^2
RECOVA symptom score*	Post-dialysis fluid overload	1.57 [0.2, 12.04]	0.81	0.59	3.92	1.12	36	25	0.56
RECOVA symptom score*	Post-dialysis blood volume	1.03 [0.21, 4.98]	0.98	0.56	8.62	1.02	43	26	0.74
Pre-dialysis blood volume	Post-dialysis fluid overload	0.08 [-0.31, 0.47]	0.83	0.92	0.91	NA	124	31	0.70
Pre-dialysis blood volume	Post-dialysis blood volume	0.52 [0.32, 0.71]	<0.001	0.23	0.50	NA	177	32	0.53
Symptomatic hypotension or cramps*	Post-dialysis fluid overload	0.38 [0.07, 1.98]	0.45	0.55	3.69	NA	124	42	0.37
Symptomatic hypotension or cramps*	Post-dialysis blood volume	2.2 [0.77, 6.27]	0.30	0.66	2.68	NA	125	36	0.30
Dialysis satisfaction*	Post-dialysis fluid overload	0.57 [0.18, 1.82]	0.57	0.61	1.14	1.45	117	39	0.25
Dialysis satisfaction*	Post-dialysis blood volume	0.92 [0.44, 1.92]	0.90	0.42	2.57	1.10	116	33	0.49
Recovery time*	Post-dialysis fluid overload	2.19 [0.67, 7.2]	0.39	0.26	15.46	2.17	120	39	0.73
Recovery time*	Post-dialysis blood volume	1.8 [0.7, 4.61]	0.42	0.37	1483.24	3.49	119	33	0.74
Pre-dialysis fluid overload	Post-dialysis fluid overload	-0.16 [-0.76, 0.43]	0.78	0.08	0.87	0.28	141	41	0.63
Pre-dialysis fluid overload	Post-dialysis blood volume	-0.47 [-0.98, 0.04]	0.19	0.03	3.87	0.71	115	29	0.63
Hypertension medications*	Post-dialysis fluid overload	1.3 [0.64, 2.66]	0.66	0.90	5774.86	NA	359	48	0.96
Hypertension medications*	Post-dialysis blood volume	0.89 [0.54, 1.49]	0.81	0.88	7378.95	NA	364	38	0.96
Intradialytic hypotension*	Post-dialysis fluid overload	0.53 [0.17, 1.68]	0.49	0.70	3.89	NA	315	48	0.39
Intradialytic hypotension*	Post-dialysis blood volume	0.91 [0.48, 1.74]	0.90	0.66	2.95	NA	339	37	0.27
Well-being*	Post-dialysis fluid overload	0.84 [0.33, 2.17]	0.87	0.81	4.23	NA	124	42	0.39
Well-being*	Post-dialysis blood volume	1.06 [0.57, 1.99]	0.92	0.42	5.84	NA	125	36	0.50

Legend to Table S12: Only data from patients with average relative fluid overload >15% during *Evaluation 1* were analyzed. Dependent variables (DV) were modelled with generalized linear mixed-effects models. Post-dialysis fluid overload and blood volume were fit as independent variables of interest (IVOI) delayed by 2-3 days in relation to the DV, with baseline age (z-score), sex, type 2 diabetes mellitus, heart failure, albumin concentration (z-score), hemoglobin concentration (z-score) and dialysis vintage (z-score) as additional fixed effects, and an interaction term between the IVOI and the time delay. "β_{ivoi} [95% CI]" denotes the effect estimates of the IVOI. Estimates marked with an asterisk (*) were reported as odds ratios, and as regression coefficients otherwise. σ_0 and σ_1 indicate the standard deviation of intercepts and slopes between patients. If σ_1 is NA, the model was fit without random slopes. Abbreviations: CI, confidence interval; DV, dependent variable; IVOI, independent variable of interest; SD, standard deviation.

Table S13: Project perception of patients and nurses

Question	N = 89
Nurses: Is the patient's fluid status adequately assessed at Check-In?	61 (69%)
Nurses: Is the patient's fluid status adequately assessed at Check-Out?	68 (76%)
Patients: Is your fluid status adequately assessed at Check-In?	52 (58%)
Patients: Is your fluid status adequately assessed at Check-Out?	67 (75%)
Patients: Were procedures for fluid status assessment too much to bear during the project?	20 (22%)

Legend to Table S13: The data are reported as frequencies and percentages of „yes" responses. Only complete cases with data from both *Check-In* and *Check-Out* were analyzed.

Supplemental Figures

Figure S1: Long-Itch report

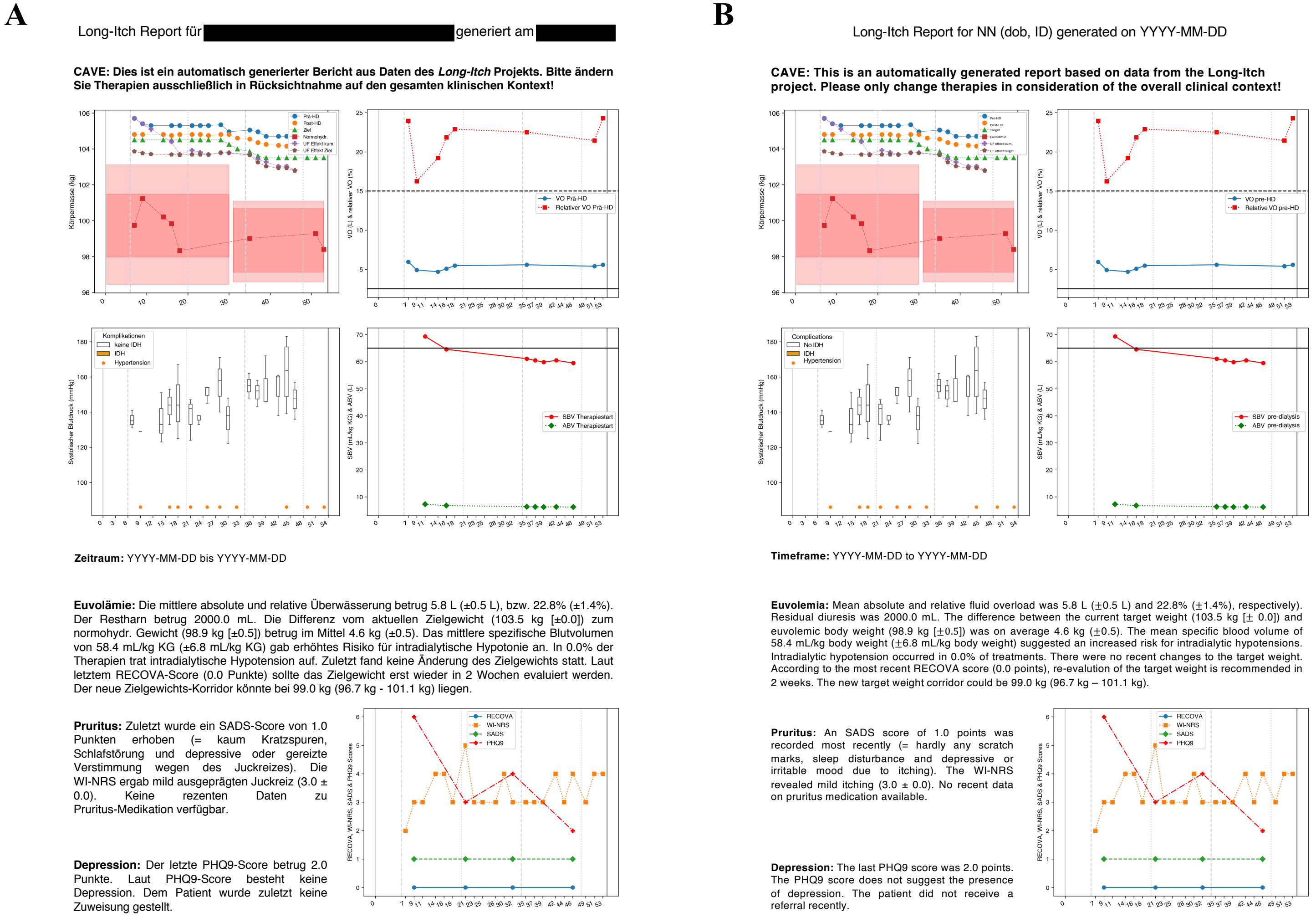
A

Long-Itch Report für [redacted] generiert am [redacted]

CAVE: Dies ist ein automatisch generierter Bericht aus Daten des *Long-Itch* Projekts. Bitte ändern Sie Therapien ausschließlich in Rücksichtnahme auf den gesamten klinischen Kontext!

Zeitraum: YYYY-MM-DD bis YYYY-MM-DD

Euvolämie: Die mittlere absolute und relative Überwässerung betrug 5.8 L (±0.5 L), bzw. 22.8% (±1.4%). Der Restharn betrug 2000.0 mL. Die Differenz vom aktuellen Zielgewicht (103.5 kg [±0.0]) zum normohydr. Gewicht (98.9 kg [±0.5]) betrug im Mittel 4.6 kg (±0.5). Das mittlere spezifische Blutvolumen von 58.4 mL/kg KG (±6.8 mL/kg KG) gab erhöhtes Risiko für intradialytische Hypotonie an. In 0.0% der Therapien trat intradialytische Hypotonie auf. Zuletzt fand keine Änderung des Zielgewichts statt. Laut letztem RECOVA-Score (0.0 Punkte) sollte das Zielgewicht erst wieder in 2 Wochen evaluiert werden. Der neue Zielgewichts-Korridor könnte bei 99.0 kg (96.7 kg - 101.1 kg) liegen.

Pruritus: Zuletzt wurde ein SADS-Score von 1.0 Punkten erhoben (= kaum Kratzspuren, Schlafstörung und depressive oder gereizte Verstimmung wegen des Juckreizes). Die WI-NRS ergab mild ausgeprägten Juckreiz (3.0 ± 0.0). Keine rezenten Daten zu Pruritus-Medikation verfügbar.

Depression: Der letzte PHQ9-Score betrug 2.0 Punkte. Laut PHQ9-Score besteht keine Depression. Dem Patient wurde zuletzt keine Zuweisung gestellt.

B

Long-Itch Report for NN (dob, ID) generated on YYYY-MM-DD

CAVE: This is an automatically generated report based on data from the Long-Itch project. Please only change therapies in consideration of the overall clinical context!

Timeframe: YYYY-MM-DD to YYYY-MM-DD

Euvolemia: Mean absolute and relative fluid overload was 5.8 L (±0.5 L) and 22.8% (±1.4%), respectively). Residual diuresis was 2000.0 mL. The difference between the current target weight (103.5 kg [± 0.0]) and euvolemic body weight (98.9 kg [±0.5]) was on average 4.6 kg (±0.5). The mean specific blood volume of 58.4 mL/kg body weight (±6.8 mL/kg body weight) suggested an increased risk for intradialytic hypotensions. Intradialytic hypotension occurred in 0.0% of treatments. There were no recent changes to the target weight. According to the most recent RECOVA score (0.0 points), re-evalution of the target weight is recommended in 2 weeks. The new target weight corridor could be 99.0 kg (96.7 kg – 101.1 kg).

Pruritus: An SADS score of 1.0 points was recorded most recently (= hardly any scratch marks, sleep disturbance and depressive or irritable mood due to itching). The WI-NRS revealed mild itching (3.0 ± 0.0). No recent data on pruritus medication available.

Depression: The last PHQ9 score was 2.0 points. The PHQ9 score does not suggest the presence of depression. The patient did not receive a referral recently.

Legend to Figure S1: A sample of the Long-Itch report in the (A) original German version and (B) translated to English.

Figure S2: Run charts

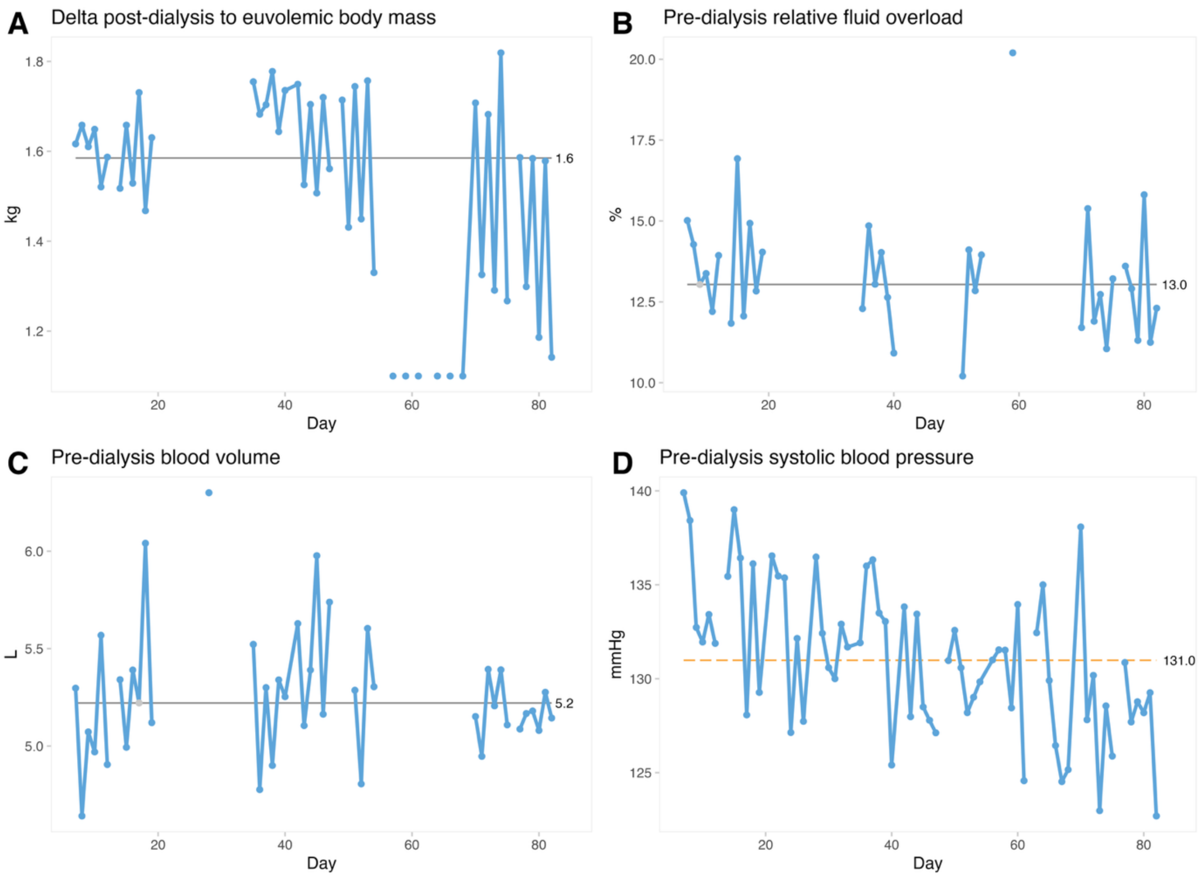

Legend to Figure S2: Panels A-D show run charts of selected variables throughout the quality improvement project.

References

1. Moissl UM, Wabel P, Chamney PW, Bosaeus I, Levin NW, Bosy-Westphal A, et al. Body fluid volume determination via body composition spectroscopy in health and disease. Physiol Meas. 2006 Sep;27(9):921–33.

2. Pietribiasi M, Waniewski J, Wójcik-Załuska A, Załuska W, Lindholm B. Model of fluid and solute shifts during hemodialysis with active transport of sodium and potassium. Idema T, editor. PLOS ONE. 2018 Dec 28;13(12):e0209553.

3. Chamney PW, Wabel P, Moissl UM, Müller MJ, Bosy-Westphal A, Korth O, et al. A whole-body model to distinguish excess fluid from the hydration of major body tissues. Am J Clin Nutr. 2007 Jan;85(1):80–9.

4. Fresenius Medical Care [Internet]. [cited 2024 Dec 1]. BCM - Body Composition Monitor. Available from: https://www.freseniusmedicalcare.com/en/body-composition-monitor
